# Sialic acids in infection and their potential use in detection and protection against pathogens

**DOI:** 10.1039/d3cb00155e

**Published:** 2023-12-19

**Authors:** Simone Dedola, Sanaz Ahmadipour, Peterson de Andrade, Alexander N. Baker, Andrew N. Boshra, Simona Chessa, Matthew I. Gibson, Pedro J. Hernando, Irina M. Ivanova, Jessica E. Lloyd, María J. Marín, Alexandra J. Munro-Clark, Giulia Pergolizzi, Sarah-Jane Richards, Iakovia Ttofi, Ben A. Wagstaff, Robert A. Field

**Affiliations:** a Department of Chemistry and Manchester Institute of Biotechnology, University of Manchester 131 Princess Street Manchester M1 7DN UK robert.field@manchester.ac.uk; b Iceni Glycoscience Ltd, Norwich Research Park Norwich NR4 7TJ UK simone.dedola@iceniglycoscience.com; c Department of Chemistry, University of Warwick Gibbet Hill Road Coventry CV4 7AL UK; d Division of Biomedical Sciences, Warwick Medical School Coventry CV4 7AL UK; e School of Chemistry, University of East Anglia, Norwich Research Park Norwich NR4 7TJ UK; f Department of Pharmaceutical Organic Chemistry, Faculty of Pharmacy, Assiut University Assiut 71526 Egypt

## Abstract

In structural terms, the sialic acids are a large family of nine carbon sugars based around an alpha-keto acid core. They are widely spread in nature, where they are often found to be involved in molecular recognition processes, including in development, immunology, health and disease. The prominence of sialic acids in infection is a result of their exposure at the non-reducing terminus of glycans in diverse glycolipids and glycoproteins. Herein, we survey representative aspects of sialic acid structure, recognition and exploitation in relation to infectious diseases, their diagnosis and prevention or treatment. Examples covered span influenza virus and Covid-19, *Leishmania* and *Trypanosoma*, algal viruses, *Campylobacter*, *Streptococci* and *Helicobacter*, and commensal *Ruminococci*.

## Abstract 2

An overview of sialic acid involvement in infection and exploitation in diagnostics and therapeutics.

## Graphical abstract

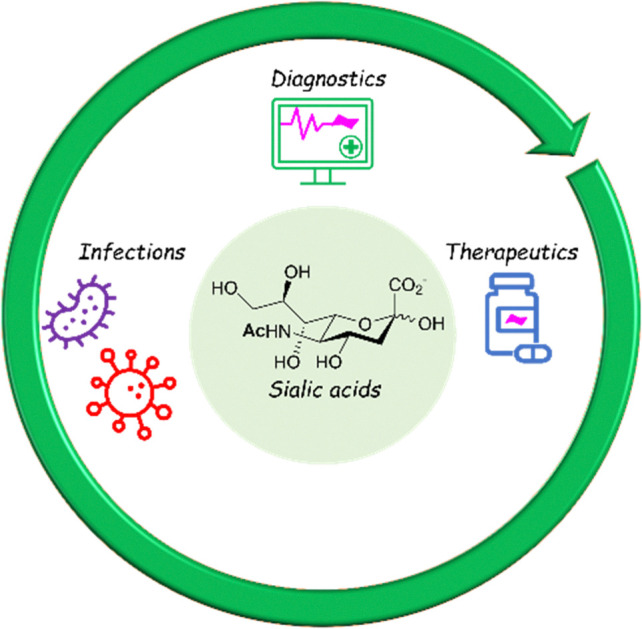

## Introduction

It is increasingly evident that carbohydrates contribute much to biology^1–5^ beyond serving as an energy source or structural material. This is particularly evident for the C9 nonulosonic acids, generally referred to as sialic acids, which are commonplace in nature.^6–10^ They are found in a number of structural forms and physiological contexts. Routinely occurring as non-reducing terminal sugar units in diverse glycan structures, sialic acids and the recognition thereof are associated with a range of health and disease scenarios, where they are intimately associated with self- and non-self-recognition. They are commonly associated with infection events – processes mediated by the interaction of microbial or viral surface protein and specific sialic acids^11–18^ found on host cell surfaces.

### Sialic acid structure and occurrence

**Fig. 1:**
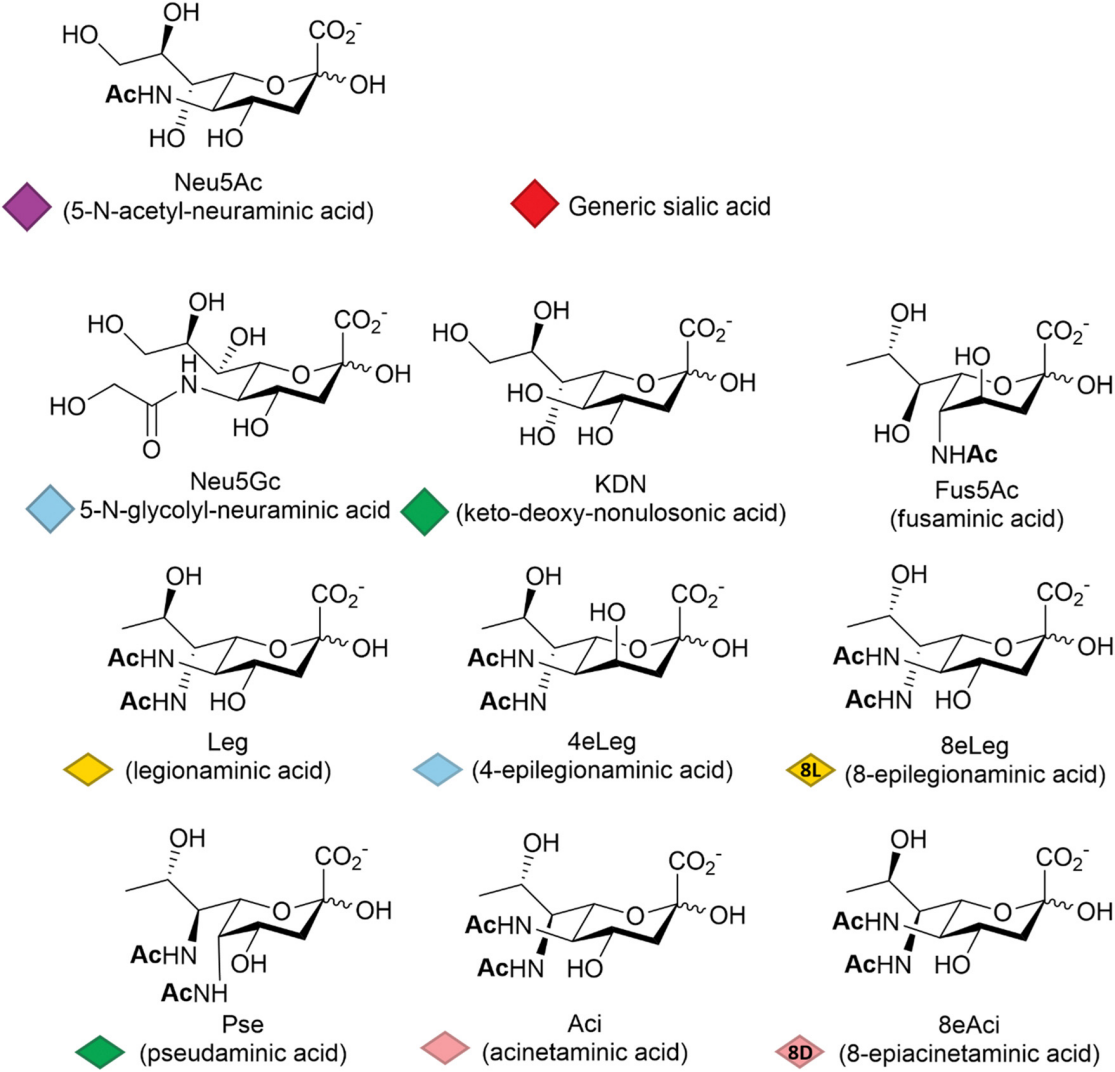
Representative sialic acid structural variants. A Symbol Nomenclature for Glycans (SNFG) has been introduced to standardise and simplify glycans drawing. The nonulosonic acids are represented by either a filled diamond shape (NeuAc, KDN *etc.*) or by a flat diamond shape (Leg, Aci *etc.*), reported below each of the corresponding chemical structure; a red filled diamond shape is used to indicate a generic sialic acid.

Among other factors, host-pathogen specificity can be determined by the large number of naturally occurring sialic acids, of which there are at least 60 different forms.^19^ Such diversity is achieved by a range of post-glycosylation modifications that involve attachment of functional groups at different sites on the main C9 skeleton. In humans, the most common modification is *N*-acetylation at position 5 (Neu5Ac, Fig. 1), while in vertebrates that have retained a functional hydroxylase-encoding gene, which has been lost in the human lineage,^20^*N*-glycolylation predominates (Neu5Gc, Fig. 1). *O*-Acetylation is found widely across species^21,22^ and can occur at positions 4, 7, 8 and 9 of the sialic acid skeleton.^23^ Other reported modifications include *O*-methylation, *O*-sulfation and *O*-phosphorylation,^19^ further expanding the structural and physicochemical diversity of the sialic acids.

The deaminated form of neuraminic acid, 2-keto-3-deoxy-d-glycero-d-galacto-nononic acid or keto-deoxy-nonulosonic acid (KDN, Fig. 1), first identified in rainbow trout eggs,^24^ is reported to occur widely among vertebrates and bacteria,^25^ while recent studies have also noted its likely widespread occurrence in microalgae.^26^ In prokaryotes, the nonulosonic acids are commonly implicated in the interaction with pathogens, being involved in the infection process and the disease development including reduced host interaction (exploiting the negative charge), altering the host immune response and molecular mimicry – thought to be a means of avoiding host immune responses. Although much better studied in vertebrates, recent years have seen variations of the sialic acid structure found in bacteria, such as fusaminic acid (Fus5Ac – the chirality of which has only been tentatively assigned),^27^ the KDN stereoisomeric legionaminic acid (Leg) and its two isomers 4-epilegionaminic acid (4eLeg) and 8-epilegionaminic acid (8eLeg), pseudaminic acid (Pse), acinetaminic acid (Aci) and its isomer 8-epiacinetaminic acid (8eAci), all reported in Fig. 1. Furthermore, the presence of Pse/Leg and KDN in samples from environmental biofilms may indicate additional overlooked roles for the sialic acids,^28^ which has consequently prompted renewed interest in the chemical diversity of these acidic sugars, including recent large scale metabolite discovery activities based on mass spectrometry methods.^29^

## Sialic acids and infection

Sialic acids are present in abundance on host organism cell surfaces as the non-reducing terminal sugar of simple glycolipids and complex glycans. As such, they are often key receptors for pathogens to adhere to host cells – a prelude to infection.^30^

### Influenza viruses, their surface proteins and sialic acid specificities

**Fig. 2:**
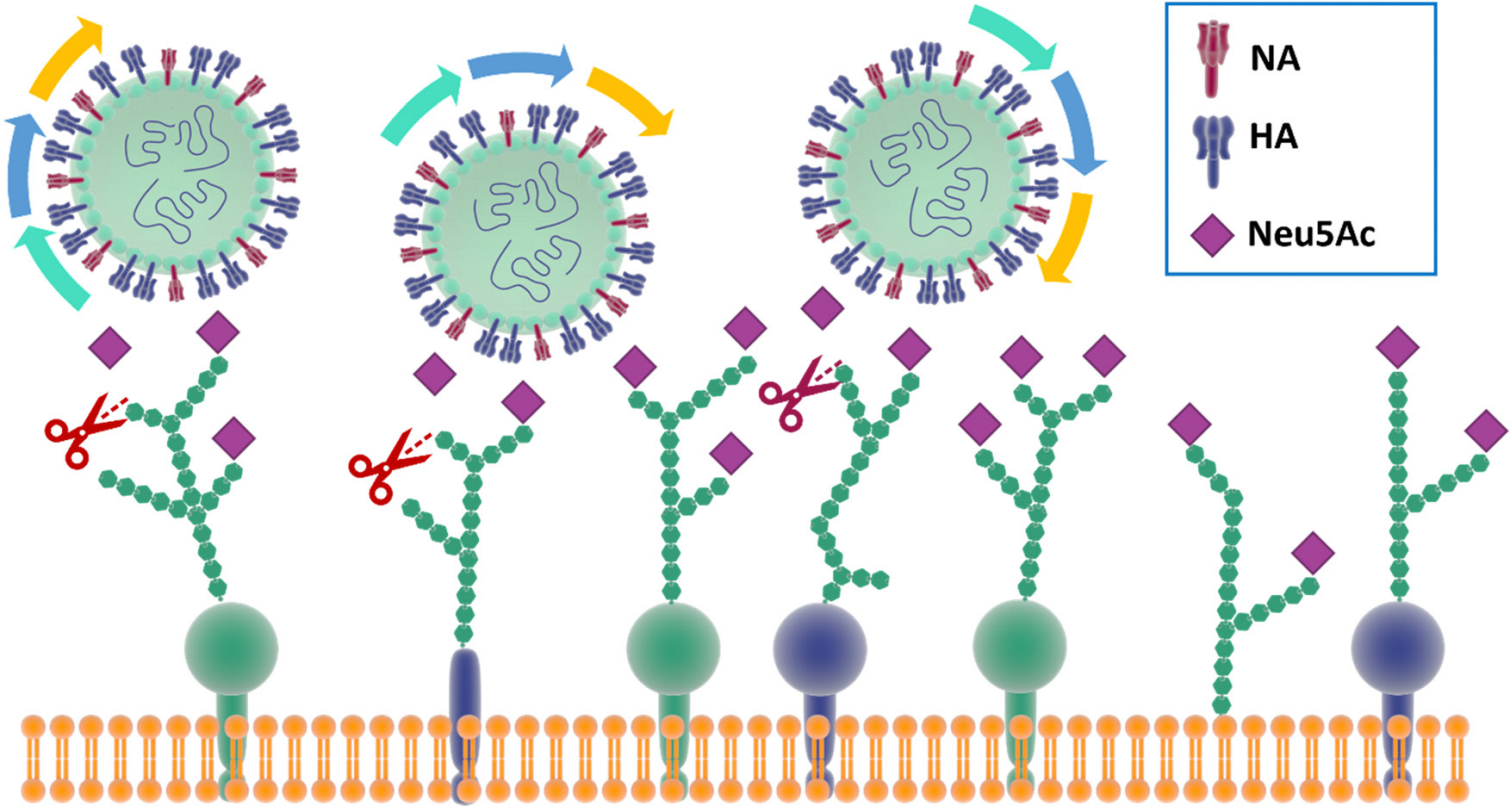
The mechanism for influenza molecular walker was firstly described by Sakai *et al.*^37^ The HAs on the influenza virus surface bind to the sialic acid on the host cell receptors with the typical carbohydrate–lectin multimeric interaction. The NA hydrolyse the sialic acid, liberating the virus from binding and triggering the “rolling” of the virus on the cell surface. The alternation of HA and NA interaction correspond to an association–disassociation events that generates the crawling and gliding motion of the virus.

In particular, and by far the most heavily studied, influenza viruses interact with Neu5Ac on the host through its haemagglutinin (HA), a trimeric protein containing the Neu5Ac receptor binding site (RBS), and neuraminidase (NA), a tetrameric protein which is responsible for cleavage of Neu5Ac. These proteins constitute the spikes through which influenza viruses can make contact and then infect their host cells.^30^ Influenza viruses have been defined as molecular walkers^31^ because of their ability to move through the thick sialic acid-containing glycan layer that covers the cells, thanks to the concerted activity^32^ of HA and NA:HA binds to the Neu5Ac receptors while NA cleaves it avoiding virus aggregation and allowing the virus to move deeper into the glycan layer until reaching the cell membrane (Fig. 2).^31^ HA generally constitutes *ca.* 80% of surface glycoproteins on influenza virus, the remaining being NA.^33^ For good viral growth, influenza viruses must have an optimal balance between HA and NA activity; any change to HA or NA activity, as a result of mutation or the presence of an inhibitor, can disturb the viral infection, replication and release cycle, and hence impact the infectivity of the virus.^32–36^

**Fig. 3:**
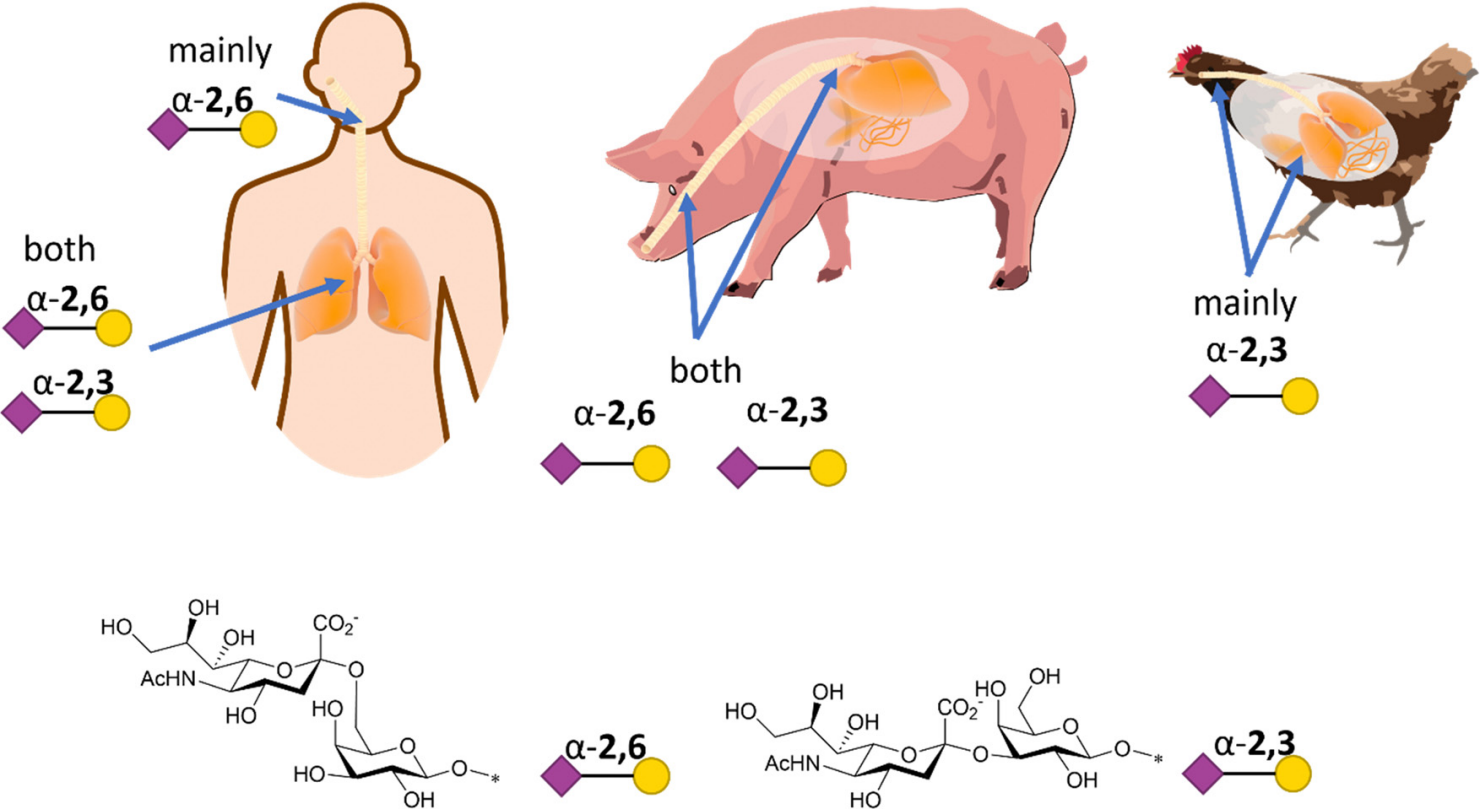
Schematic representation of α-2,6- and α-2,3-Neu5Ac-Gal receptors in humans, pigs, and chicken with structure and SNFG representation (based on De Graaf *et al.*^38^).

Influenza HA binds not only to terminal Neu5Ac but also to part of the underlying glycan to which Neu5Ac is attached. Indeed, influenza viruses discriminate between prospective hosts through binding with specific sialylated oligosaccharides structures.^38^ This specificity reflects the predominant glycan composition of the host species. For instance, human influenza viruses bind preferentially to α-2,6-Neu5Ac-Gal receptors, which are prevalent in the human upper respiratory tract (Fig. 3).^38^ On the other hand, avian influenza viruses bind preferentially to α-2,3-Neu5Ac-Gal receptors, with avian species expressing mainly α-2,3-Neu5Ac-Gal receptors in the respiratory tract (Fig. 3).^38^ Pigs, on the other hand, express both α-2,6 and α-2,3-Neu5Ac-Gal receptors in their respiratory tract, can be infected with both human and avian influenza viruses and have been consequently defined as “mixing vessels” for virus reassortment among avian, swine and human.^38^ Horses and pigs predominantly express the glycolyl form of sialic acid, as α-2,3-Neu5Gc-Gal, in their trachea. Given that influenza A viruses can be strictly selective toward Neu5Ac or Neu5Gc, this presents a species jump barrier, given the inability of humans to biosynthesise Neu5Gc.^39^

**Fig. 4:**
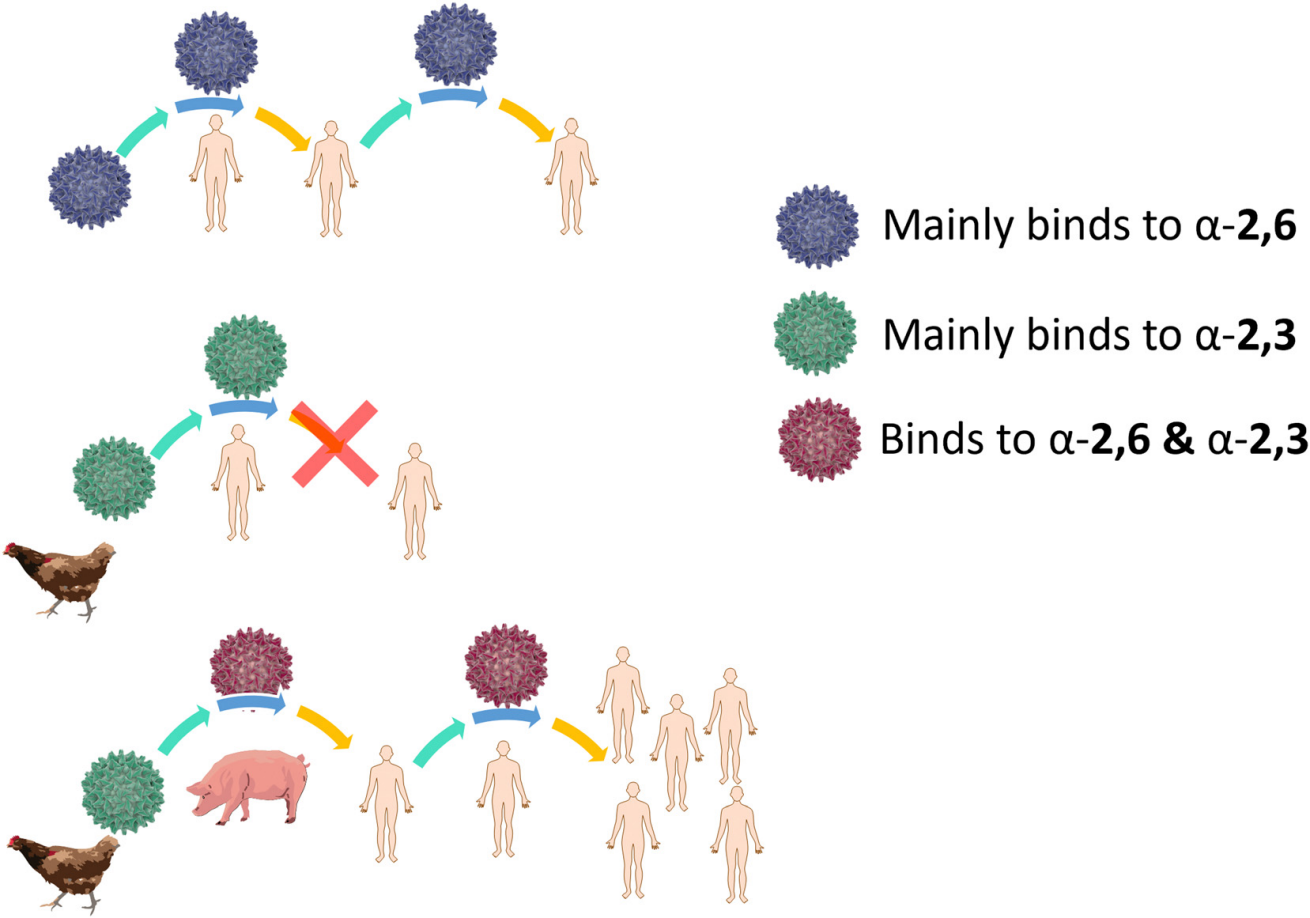
Human flu virus binds mainly α-2,6-Neu5Ac-Gal, it can infect humans and can be transmitted (top). Avian virus binds mainly α-2,3-Neu5Ac-Gal, it can infect humans if reaches the lower respiratory tract, where the α-2,3-Neu5Ac-Gal is present, however transmission to other individuals is difficult (middle). Avian virus that infects pigs can switch to α-2,6-Neu5Ac-Gal binding, infect humans and potentially cause a pandemic (bottom).

For an animal virus to cross the species barrier and infect humans, the virus must be able to bind to both the animal and human sialic acid receptors (Fig. 4). This has been demonstrated for a variety of avian viruses, such as H1N1, H3N2, H5N1 and H7Nx, which have HA mutations that switch its preference from α-2,3-Neu5Ac-Gal to α-2,6-Neu5Ac-Gal.^40^ However, while receptor specificity is a requirement to cross the species barrier, not all animal viruses can spread between humans by airborne transmission and become pandemic. While it is generally accepted that only viruses with α-2,6-Neu5Ac-Gal affinity transmit efficiently between humans, other factors involved in the airborne transmission are not yet completely understood. The stability of HA mutants, the HA/NA balance, and the efficiency of polymerase-mediated replication are all factors that may contribute to virus adaptation to their new host species.^38^

**Fig. 5:**
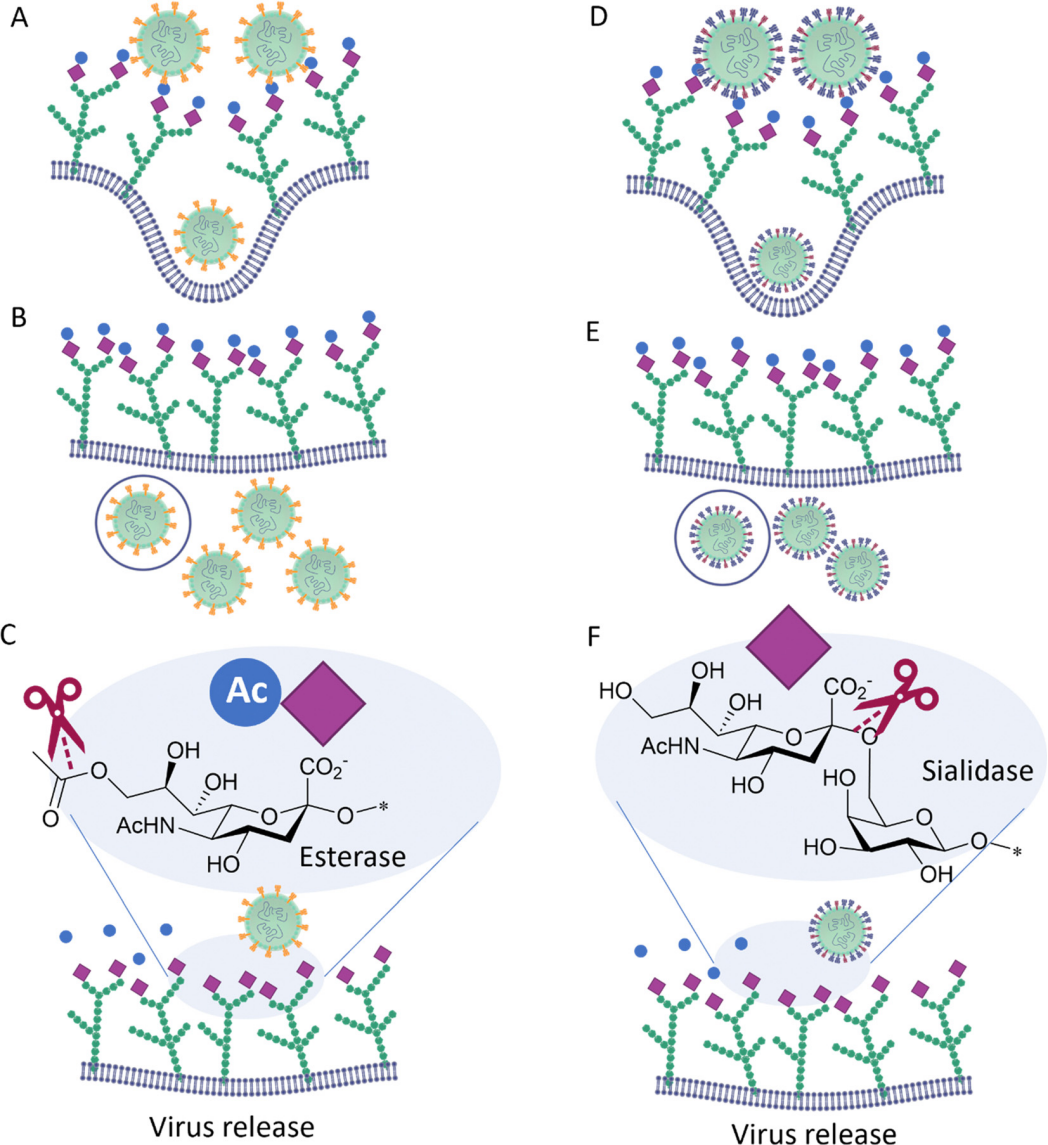
The Hemagglutinin–Esterase-Fusion surface protein on influenza C virus (left) surface binds to the glycans on host cell surface and the virus is internalised (A), replicated (B) and released (C) outside the cells, facilitated by the esterases action that destroy the binding glycan moiety. The same function is performed by the two different surface proteins HA and NA in influenza A and B viruses (right), D, E and F.

Influenza C, in contrast to influenza A and B, possesses only one surface protein, designated Hemagglutinin–Esterase-Fusion (HEF) protein, that has HA and NA activity, as well as an esterase function.^31^ HEF recognizes 9-*O*-acetyl-*N*-acetylneuraminic acid (Neu5,9Ac_2_) and acts as a receptor-destroying enzyme by selectively removing the 9-*O*-acetyl group (Fig. 5). Similar to influenza C HEF, some coronaviruses (see below) have evolved to specifically recognize 9-*O*-acetyl-*N*-acetylneuraminic acid receptors utilizing a spike protein^31^ and to facilitate release of viral progeny *via* the sialic acid *O*-acetyl esterase activity of their Haemagglutinin–Esterase (HE).

### Coronaviruses and sialic acid recognition

Coronaviruses^13,41^ cause a range of diseases and symptoms that differ between vertebrate host species. Divided in different subfamilies, α-, β-, γ-, and δ-coronavirus, they have different affinities for Neu5Ac and its derivatives.^41^ For example, transmissible gastroenteritis virus (TGEV) and porcine respiratory coronavirus^41^ (PRCoV) are both α-coronaviruses; the former (TGEV)^42^ shows binding to Neu5Ac with preference for α-2,3-linkages, the latter (PRCoV)^41^ does not have a sialic acid binding receptor. Human coronavirus41 HCoV-299E and HCoV-NL63,^44^ both belong to the α-coronavirus family, and they appear to lack a specific sialic acid receptor, although NL63 uses heparan sulfate as an attachment factor to host cells, highlighting once more the pivotal role of virus-host carbohydrate binding in viral infections. The remaining known human^45^ coronaviruses all belong to the β-coronavirus group, showing different specificity towards sialic acid. HCoV-OC43 and HKU1^46–48^ show preferential binding to Neu5,9Ac_2_ and possess an *O*-acetyl esterase activity on their surface. The mechanism is similar to the receptor-destroying binding of influenza C described in Fig. 5 with the virus removing the 9-OAc group to facilitate release of daughter virions. The Middle East respiratory syndrome coronavirus (MERS-CoV),^49^ which emerged in 2012, belongs to the β-coronavirus family and showed binding to sialic acid, with a preference for α-2,3- over α-2,6-linked glycans.^49,50^ Interestingly, the SARS-CoV1 that emerged in 2002 belongs to the β-coronavirus subfamily, but does not have a sialic acid binding receptor. The more recently occurring SARS-CoV2, responsible for the COVID-19 related pandemic, shares many similarities with SARS-CoV1, but it has been demonstrated to bind sialic acid.^50–53^ A summary of the sialic acid receptors of coronavirus is reported in the Table 1.^41^

**Table 1:** Summary of coronavirus subfamilies and identified sialic acid and its derivatives receptors^41^

Group	Species	Protein receptor	Sialic acid receptors
α-CoVs	Transmissible gastroenteritis coronavirus (TEGV)	APN	Neu5Ac & Neu5Gc
	Canine coronavirus	APN	—
	Porcine respiratory coronavirus (BCoV)	APN	—
	Feline coronavirus (FeCoV)	APN	α-2,3/α-2,6-linked sialic acid
	Porcine Epidemic diarrhoea coronavirus (PEDV)	APN	Neu5Ac
	Human coronavirus 229E (HCoV-229E)	APN	—
	Human coronavirus NL63 (HCoV-NL63)	ACE	—

β-CoVs	Bat coronavirus (HCoV-229E)	—	Neu5,9Ac_2_
	Porcine hemagglutinating encephalomyelitis virus (PHEV)	—	Neu5,9Ac_2_
	Murine hepatitis virus	CEACAMI	Neu5,9Ac_2_, Neu4,9Ac_2_
	Human coronavirus 4408 (HCoV-4408)	—	—
	Human coronavirus OC43 (HCoV-OC43)	—	Neu5,9Ac_2_
	Human coronavirus HKUI (HCoV-HKUI)	—	Neu5,9Ac_2_
	Severe acute respiratory syndrome coronavirus (SARS-CoV)	ACE2	α-2,3/α-2,6-linked sialic acid
	Severe acute respiratory syndrome coronavirus (2019-CoV)	ACE2	α-2,3/α-2,6-linked sialic acid
	Middle Eastern respiratory syndrome coronavirus (MERS-CoV)	DPP4	α-2,3/α-2,6-linked sialic acid

γ-CoVs	Avian infectious bronchitis virus (IBV)	—	Neu5Ac
	Turkey coronavirus	—	—

The structural basis for sialic acid recognition by human coronaviruses^43^*via* surface glycoproteins has established the basis for 9-*O*-acetyl-sialoglycan engagement. The spike protein architecture is similar to that of the ligand-binding pockets of coronavirus hemagglutinin esterases and influenza virus C or D hemagglutinin–esterase fusion glycoproteins. It appears that coronavirus hemagglutinin–esterase and spike proteins have co-evolved to balance and optimise virion avidity.^54^ Above and beyond the abundant mucin glycan-based respiratory receptors for SARS-CoV2, recent studies have established that sialic acid-containing glycolipids also have the potential to mediate cell binding and viral entry.^55^

### Algae-virus interactions and KDN

**Fig. 6:**
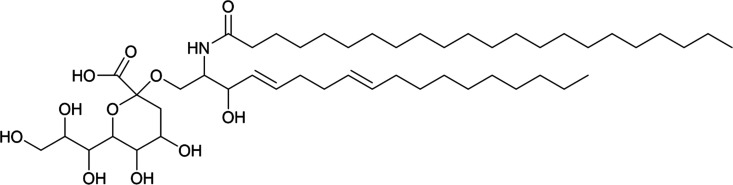
Tentative chemical structure of the novel sialic acid glycosphingolipid.^59^

Looking beyond medicine, sialic acid recognition is also evident in the wider environment. For instance, KDN has emerged as a potential key player in viral infection of KDN-producing eukaryotic microalgae^56–59^ and it is thought to play a role in harmful algal bloom dynamics. A potential KDN-containing glycosphingolipid (the stereochemistry of the sugar was not determined; a tentative structure is represented in Fig. 6) has been reported in lipid rafts from the bloom-forming microalga *Emiliania huxleyi*,^58^ which have since been shown to determine the level of susceptibility to lytic viral infection by strains of the giant *E. huxleyi* Virus (EhV).^59^

These studies showed that for all eleven *E. huxleyi* strains tested, there was a direct relationship between levels of the KDN-like glycoconjugate and susceptibility to viral infection, suggesting that KDN plays an important role in host–pathogen interactions, as seen for other sialic acids in vertebrate infection. Furthermore, recent work has reported the presence of KDN and a dedicated biosynthetic pathway for cytidine-5′-monophospho–KDN (CMP–KDN) biosynthesis in *Prymnesium parvum*,^57^ a haptophyte relative of *E. huxleyi*. Phylogenetic analyses suggest that all algae of the *Haptophyceae* and *Alveolata* phyla have these biosynthetic capabilities.^57^ Having previously discovered a giant virus that infects this alga, *P. parvum* DNA Virus (PpDNAV-BW1),^56^ and a boom in the discovery of similar giant viruses that infect microalgae, it is tempting to speculate on a broader role for KDN in algae-virus infections – one with potentially wide impact for brackish inland waterways as well as coastal regions.^60^

### Parasitic protozoan *Trypanosoma cruzi* and Neu5Ac

**Fig. 7:**
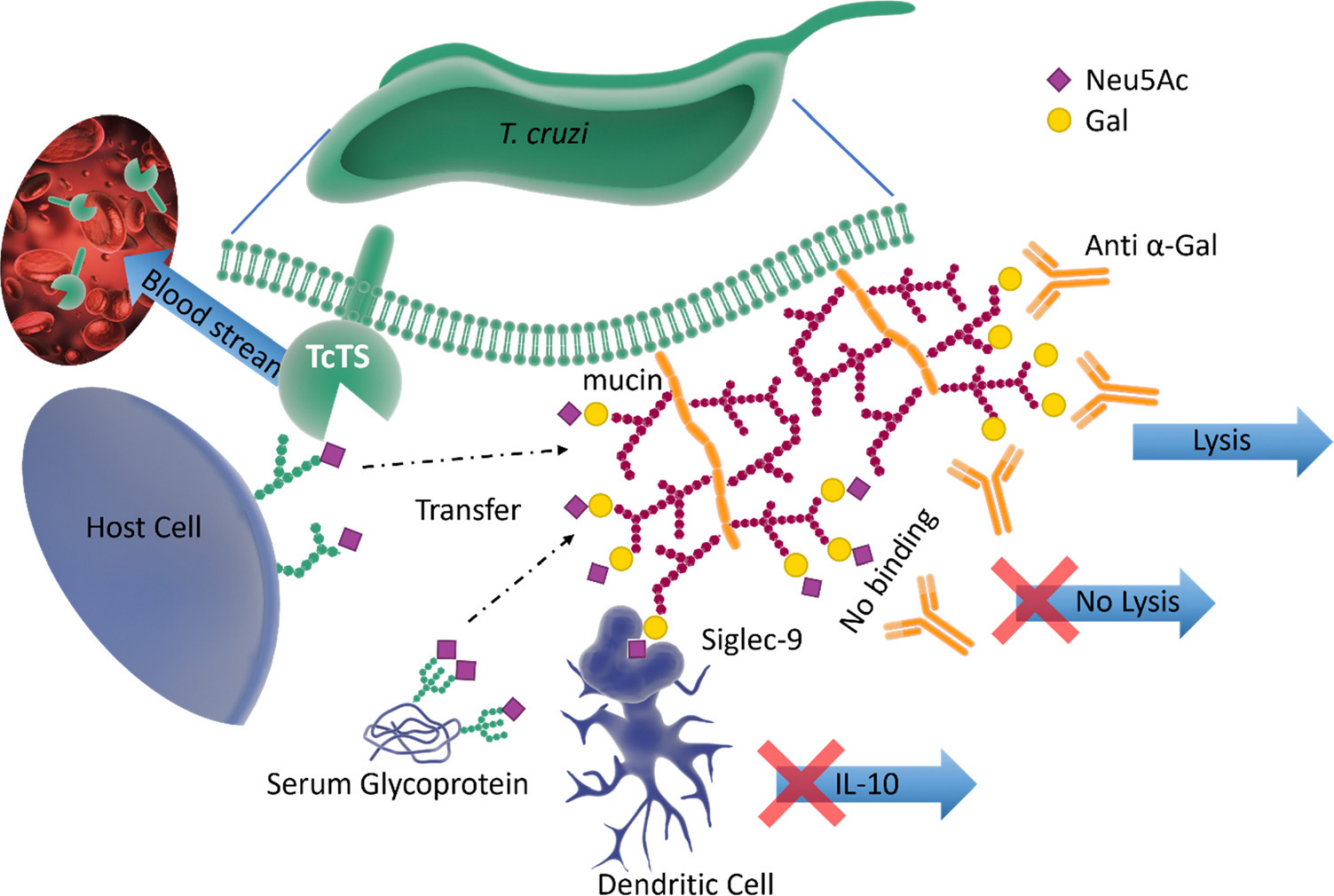
The surface of *T. cruzi* is covered with mucin containing *O*-linked glycans. The TcTS transfers sialic acid from the host cells surface glycans and serum glycoproteins to the terminal glycan residues of mucin, shielding the parasite from anti α-Gal antibodies. The newly sialylated mucin interacting with Siglec-9 on dendritic cells surface can result in suppressing the release of IL-10. TcTS is released in the blood stream where it alters the glycosylation pattern of surface proteins making the host more susceptible to infections and diseases.^64,65^

Pathogen–host interactions are often based on well-defined carbohydrate binding events. When Neu5Ac was found in the kinetoplastid parasite *Trypanosoma cruzi*,^61^ the etiologic agent of Chagas’ disease, it was accompanied by the discovery of a unique *trans*-sialidase enzyme (TcTS) associated with the parasite cell surface.^62,63^ TcTS, a CAZy family GH33 glycoside hydrolase, is attached to the parasite by a glycosylphosphatidylinositol (GPI) anchor. This multifunctional enzyme, considered the major *T. cruzi* virulence factor, has a central role in both the parasite infection process and modulation of the host immune response towards the parasite.^64^ Though *T. cruzi* is unable to synthesise Neu5Ac, this key monosaccharide is incorporated into the parasite surface due to the ability of TcTS to transfer terminal Neu5Ac from host glycoconjugates onto its GPI-anchored mucins, generating α-2,3-linked sialylated β-galactopyranose units (Fig. 7).

**Fig. 8:**
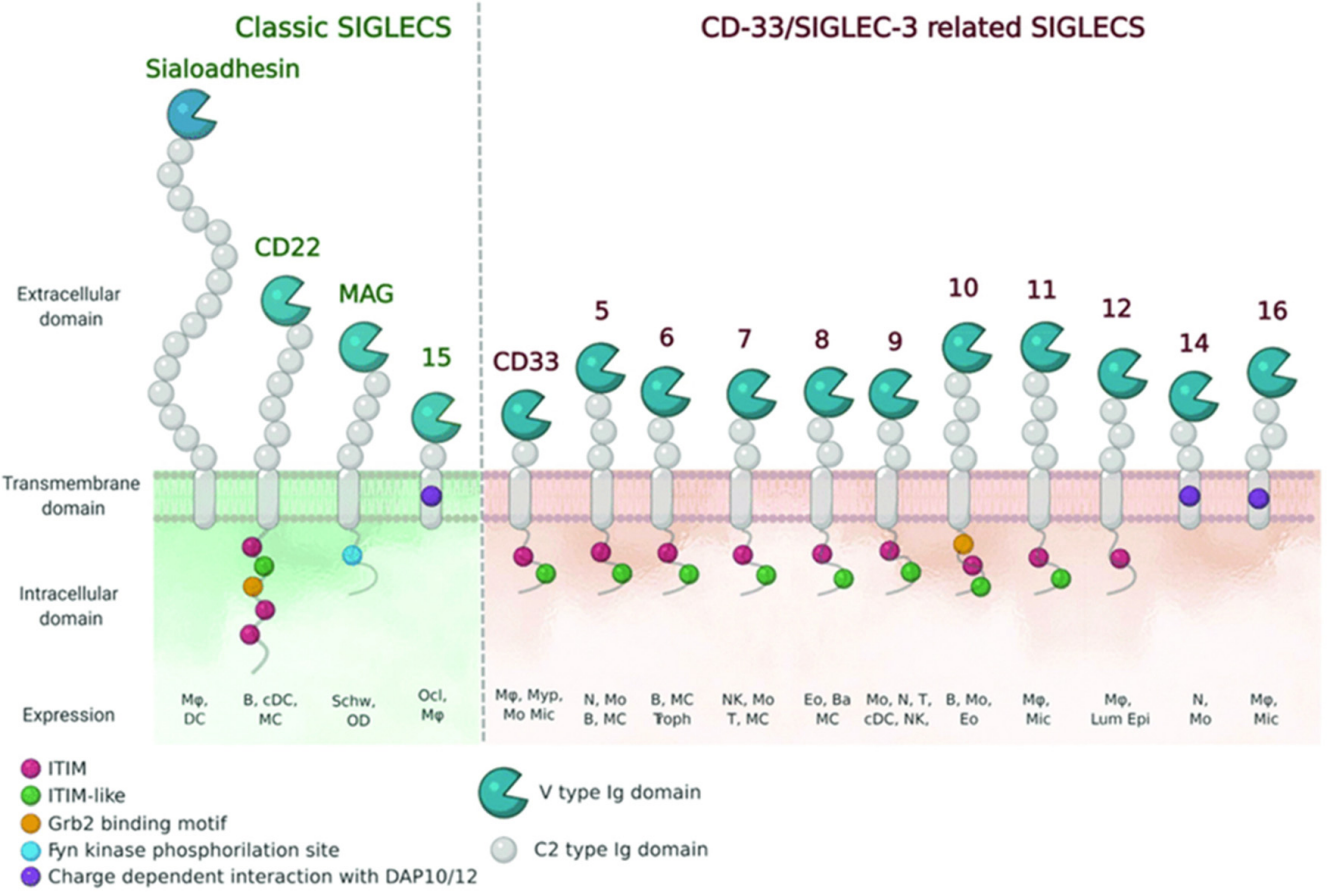
Schematic representation of Siglecs.^72^ Human Siglec receptors contain one N-terminal V-set Ig domain that is responsible for sialic acid binding and several C2-type Ig-like domains acting as spacers and determining the mode of interaction. Siglecs with ITIM (magenta) motifs are inhibitory proteins, whereas Siglecs containing ITAM (purple) motifs are activating receptors, interacting with activation partners DAP10/12. [Figure and caption reproduced from ref. Lenza *et al.*^72^ from open access MDPI, copyright 2020.]

It is known that the sialylated mucins contribute directly to the parasite adhesion and invasion of host cells, but the underlying molecular mechanism has not been elucidated.^66^ On the other hand, the negatively charged mucin coat of the parasite serves as a shield to protect the infective form of *T. cruzi* against lysis induced by host anti-α-galactosyl antibodies (Fig. 7).^65^ In mice, the sialylated mucins also interact with Neu5Ac-binding lectin-E also, Siglec-E (sialic acid-binding ImmunoGlobulin-like LECtins – Fig. 8) on host dendritic cells and triggers the suppression of cytokine interleukin 12 (IL-12), the key cytokine in the activation of the immune response.^67^ A similar mechanism may be associated with Siglec-9 and the production of IL-10 in infected humans.^68^ The parasites battle to survive and establish a persistent infection is also accompanied by the shedding of TcTS from the parasite surface into the host bloodstream, where it remodels host cell surface sialylation patterns (Fig. 7).^69^ This can induce dramatic changes in signalling and responses of targeted cells, thus enhancing host vulnerability to infection and disease.

### The sialic acid-binding lectins of the immune system

The sialic acids represent Self-Associated Molecular Patterns (SAMPs), which are recognized by inhibitory receptors with the objective to diminish unwanted immune reactions.^70^ These immune modulations can be mediated through interactions of Siglecs with sialylated glycoconjugates. The Siglec family, which includes 14 active members in humans, are type I transmembrane proteins containing an extracellular N-terminal V-set immunoglobulin (Ig) domain that is responsible for sialic acid recognition followed by a variable number (1 to 16) of so-called C2-type Ig-like domains that act as spacers, leading the ligand binding site away from the surface (Fig. 8).^71,72^ The number of C2-type domains determines the mode of interaction with sialic acid-containing glycans.

In most cases, sialic acid interacts with a Siglec on the same cell surface in *cis*-mode, whereas Siglec-1, for example, binds sialoglycans *in trans*, *i.e.* on adjacent cells.^73^ As a result, *in cis* interactions dominate over interactions with *trans* ligands, without precluding binding of ligands *in trans*. As such, the Siglecs are integral to maintaining immune homeostasis. However, they also serve to sense pathogen-associated sialic acids, but equally can represent potential vulnerability for the host where pathogens sialyated glycans are concerned. The interplay between Siglecs and sialylated pathogens^74^ – bacterial, viral and protozoan – represents an emergent field. It is expected to gain substantial momentum as we better understand how inhibitory Siglec–sialic acid interactions balance immunological activation and tolerance during viral infections,^75^ the role of Siglecs in host defense and dissemination of enveloped viruses,^76^ and infectious diseases more broadly,^75^ including bacteria-induced sepsis^77^ and infection associated with parasitic protozoa, such as *Leishmania*.^78^

### Sialic acids in *Campylobacter jejuni* lipooligosaccharide and auto-immune impacts

Polysaccharides on bacterial surfaces are often implicated in molecular mimicry of host carbohydrate structures,^79^ with sialic acids such as Neu5Ac, Neu5Gc, Pse and Leg playing a crucial albeit not fully understood role.^80–82^ Progress in this field has been reviewed recently by Wennekes *et al.*^83^ The pathogen exploits such host similarity as a camouflage to evade innate and adaptive immune system surveillance, but this molecular mimicry can cause abnormal autoimmune responses in the host, resulting in the generation of auto-antibodies and T cells that attack host tissues.^84^

**Fig. 9:**
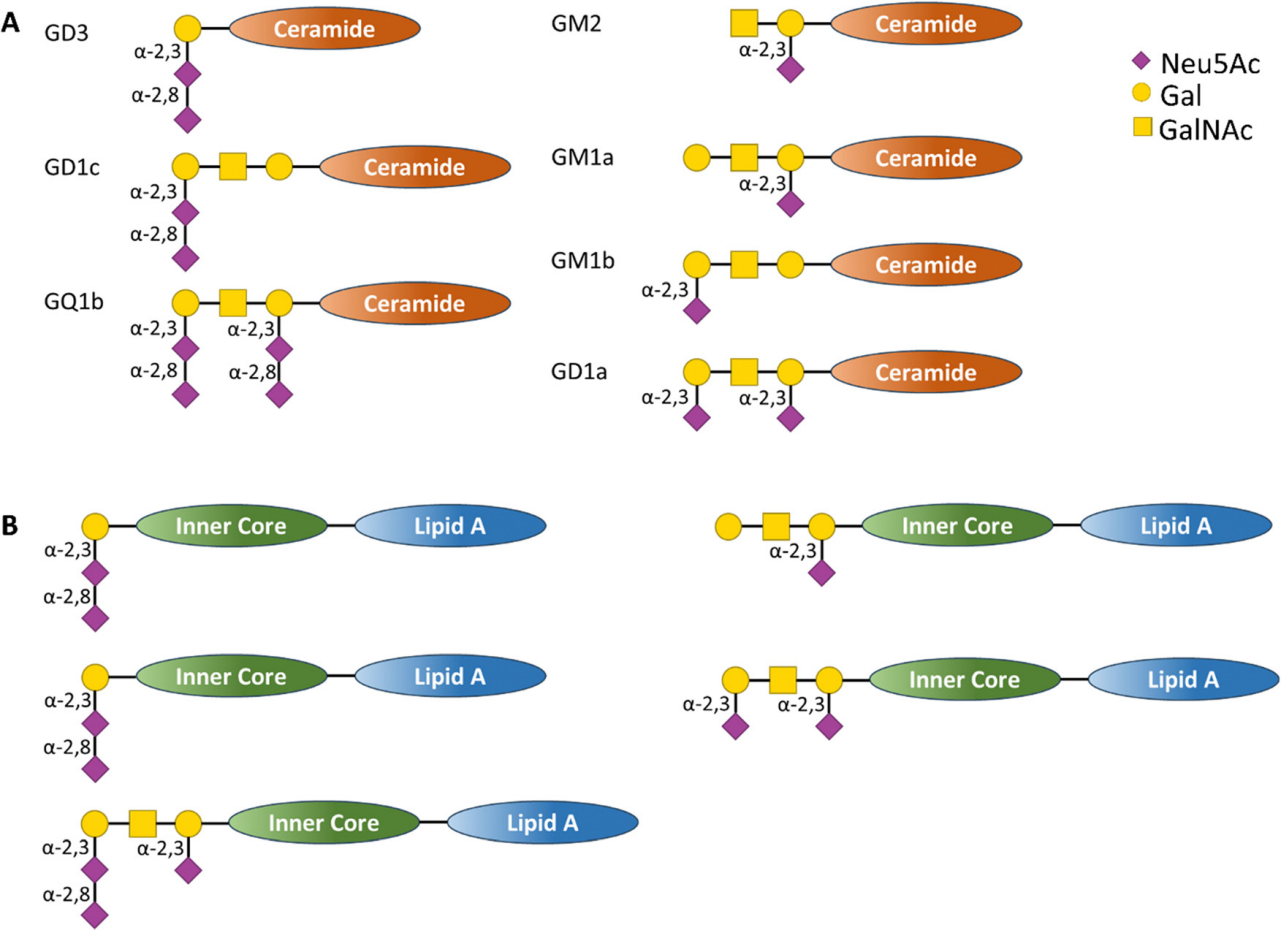
(A) Schematic representation of human ganglioside structure containing sialic acid residues bound to a ceramide inner core and (B) schematic representation of *C. jejuni* LOS structures containing sialic acid derivatives that act as structural mimic of the human ganglioside (A), in this case the glycan derivatives are bound to an inner core and lipid A transmembrane tail.^86^

The Gram-negative bacterium *Campylobacter jejuni* is the major cause of bacterial gastro-enteritis worldwide.^85^ Infection with *C. jejuni* can lead to neurological complications, including Guillain-Barré syndrome (GBS) – an immune-mediated disease affecting the peripheral nerves of the host. The relationship between *C. jejuni* and GBS has been extensively investigated. The lipo-oligosaccharide (LOS) on the outer surface of the bacteria mimic host cells Neu5Ac-containing ganglioside glycolipid structures (Fig. 9,) which are abundantly expressed on the nervous systems tissues.^86^

**Fig. 10:**
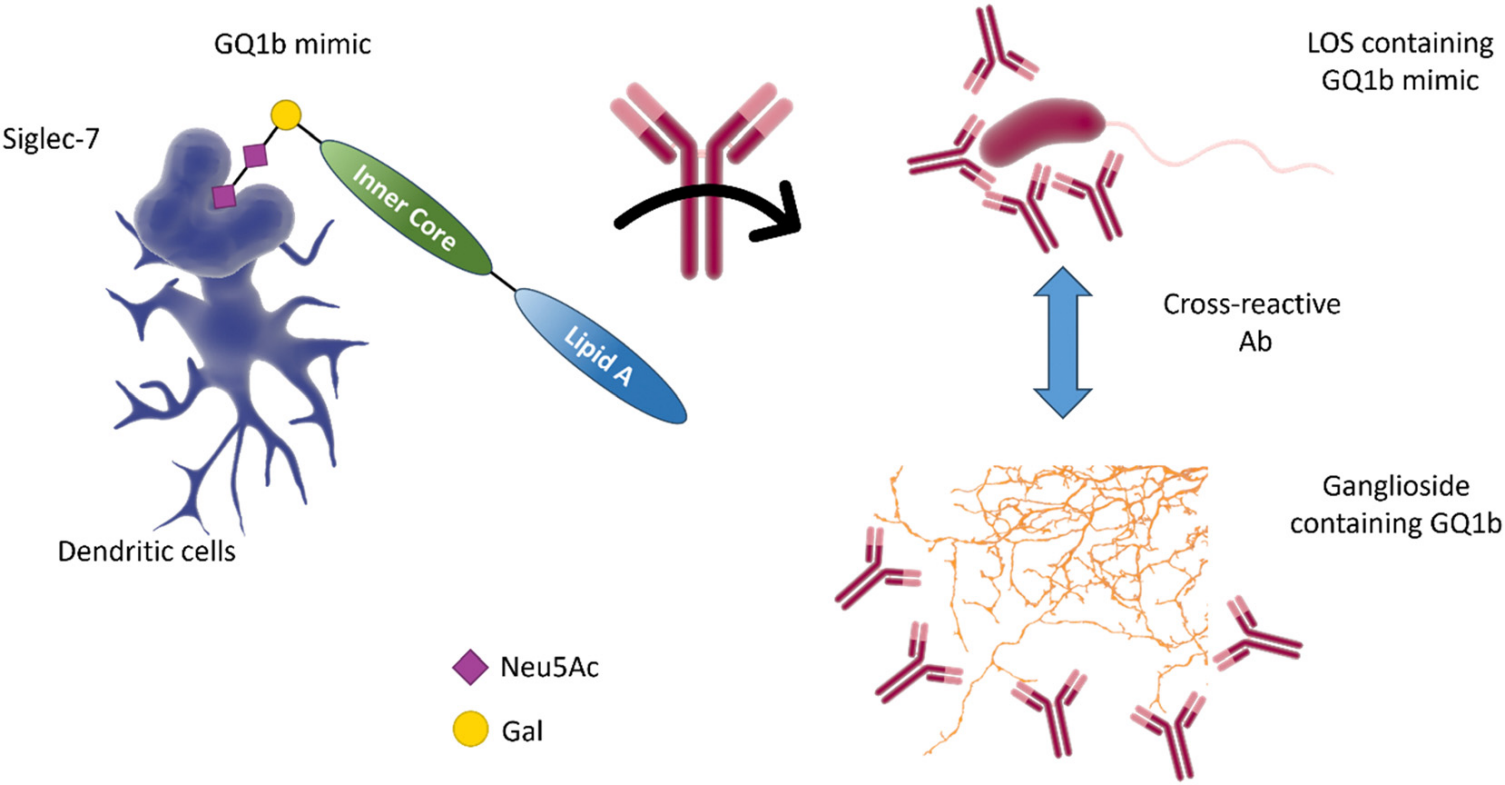
The interaction of Siglec-7 with *C. jejuni* strains expressing disialylated LOS^86^ may be related to an anti-GQ1b cross-antibody activation, leading to oculomotor weakness in patients with Guilliam-Barré syndrome or the related Miller Fisher syndrome.^88^ GQ1b disialylated structures are contained in ganglioside of the human peripheral nervous system.

The molecular mimicry between *C. jejuni* LOS and host gangliosides leads to the formation of cross-reactive antibodies directed against the peripheral nerves of the host. GBS-associated *C. jejuni* strains bind to Siglec-7,^86^ demonstrating that a sialic acid receptor is associated with inflammatory and autoimmune disease (Fig. 10).^86^ Mass spectrometry analysis demonstrated that the binding was sialic acid-linkage specific, with a preference for α-2,3-linked sialic acid attached to the terminal galactose of the LOS chain, as observed in several gangliosides (*e.g.* GD1a, GM1b, and GM3).^87^ Reports also indicate the interaction of Siglec-7 with *C. jejuni* LOS, especially with strains expressing a di-sialylated ganglioside mimic with α-2,3 or α-2,3/α-2,8 linkages.^88^ Serological studies using anti-ganglioside antibodies from GBS patients show that they recognise the LOS of *C. jejuni*, suggesting that they may have been induced by the *C. jejuni* infection.^89^

## Potential applications of sialic acids in pathogen detection

The pivotal role of sialic acid and its derivatives in infection^90^ (viruses, bacteria, protozoa) discussed in the previous section brings attention to how these glycans could be exploited as a tool to develop new methods for detection. Landa *et al.*^91^ developed a colorimetric assay for the detection of specific strains of *Staphylococcus aureus and Pseudomonas aeruginosa*, however the majority of the examples reported in literature focus on the detection of viruses, which will be the main focus of the following section.

### Neu5Ac binding, virus detection and strain discrimination

The diagnosis of influenza infection is commonly based on nucleic acid-based technologies, such as RT-PCR, or antibody-based technologies applied for instance on lateral flow devices.^92^ However, both techniques have disadvantages, including cost, the need for specialist equipment, or the need to generate new antibodies to detect emerging strains. The specificity of HA–sialic acid binding can be exploited for alternative technologies in the diagnostic field, including simple agglutination assays and more comprehensive glycan arrays, which may be used to predict the infectiousness and species specificity of a given virus dependent on its glycan binding specificity.

**Fig. 11:**
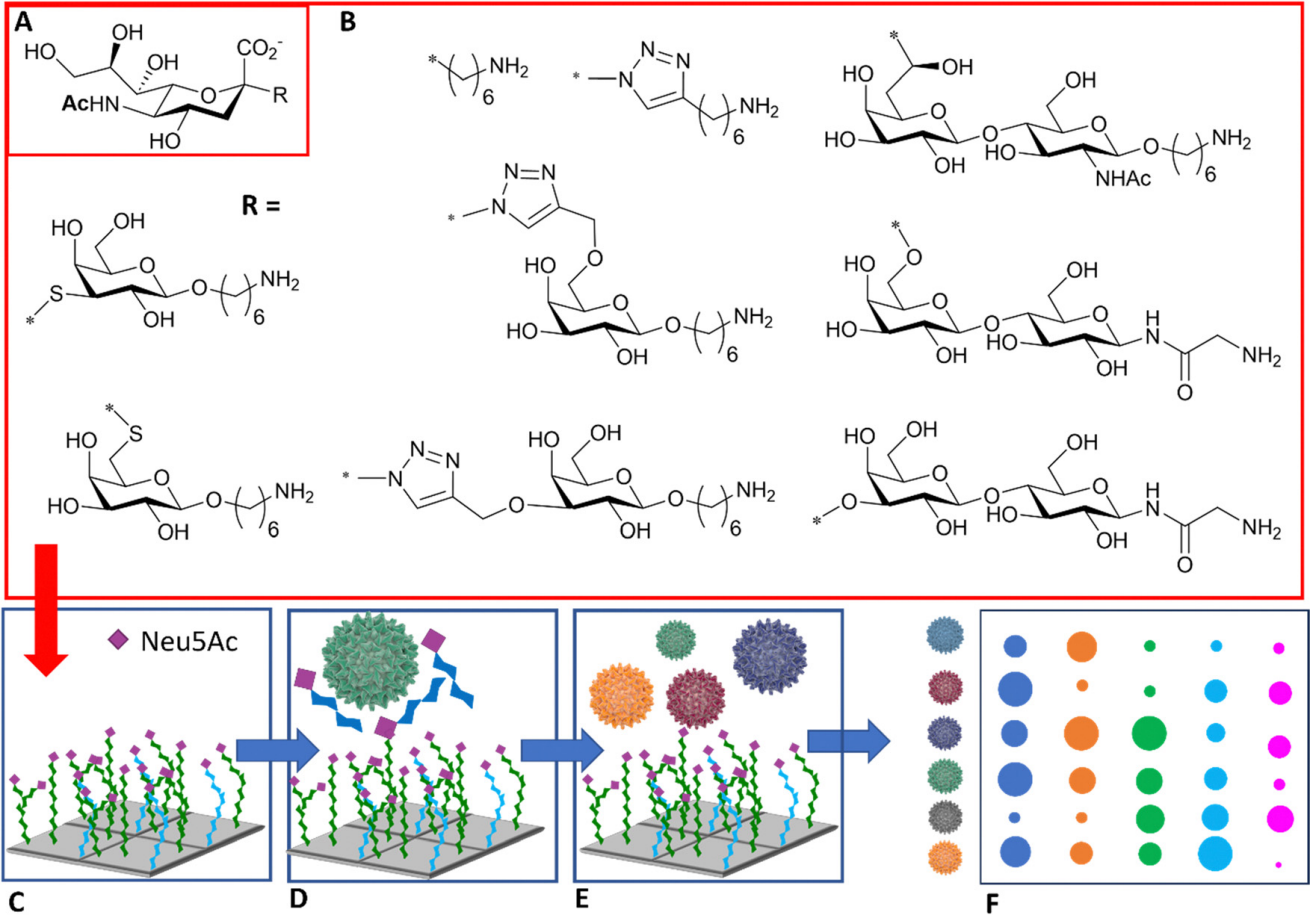
(A) Sialic acid core equipped with uncleavable linker; (B) substituent of the sialic acid core; (C) library is printed in the array; (D) binding to the viruses is inhibited in the presence of NA/HA inhibitor; (E) influenza viruses of different strains are assayed against the glycan array; (F) virus of different strains react differently with each glycan generating a signal intensity fingerprint that can be used to characterise the virus/strain.^95^

The use of high information content glycoarrays to assess HA and intact influenza virus glycan specificity is well documented and provides invaluable underpinning information for the discrimination between viral strains.^93,94^ A more focussed glycan array with potential diagnostic applications was developed by Iyer and co-workers,^95^ based on a range of *C*-, *S*-, and triazole-linked, monomeric sialosides, designed and demonstrated to resist influenza NA action (Fig. 11(A) and (B)). The authors showed that these sialosides were stable to NA and could bind intact viruses at room temperature without the need for adding NA inhibitor. Furthermore, good sensitivity and distinct fingerprint binding patterns were observed (Fig. 11(C)–(F)).^95^

### Sialylglycan-magnetic nanoparticle pull-downs for PCR analysis

**Fig. 12:**
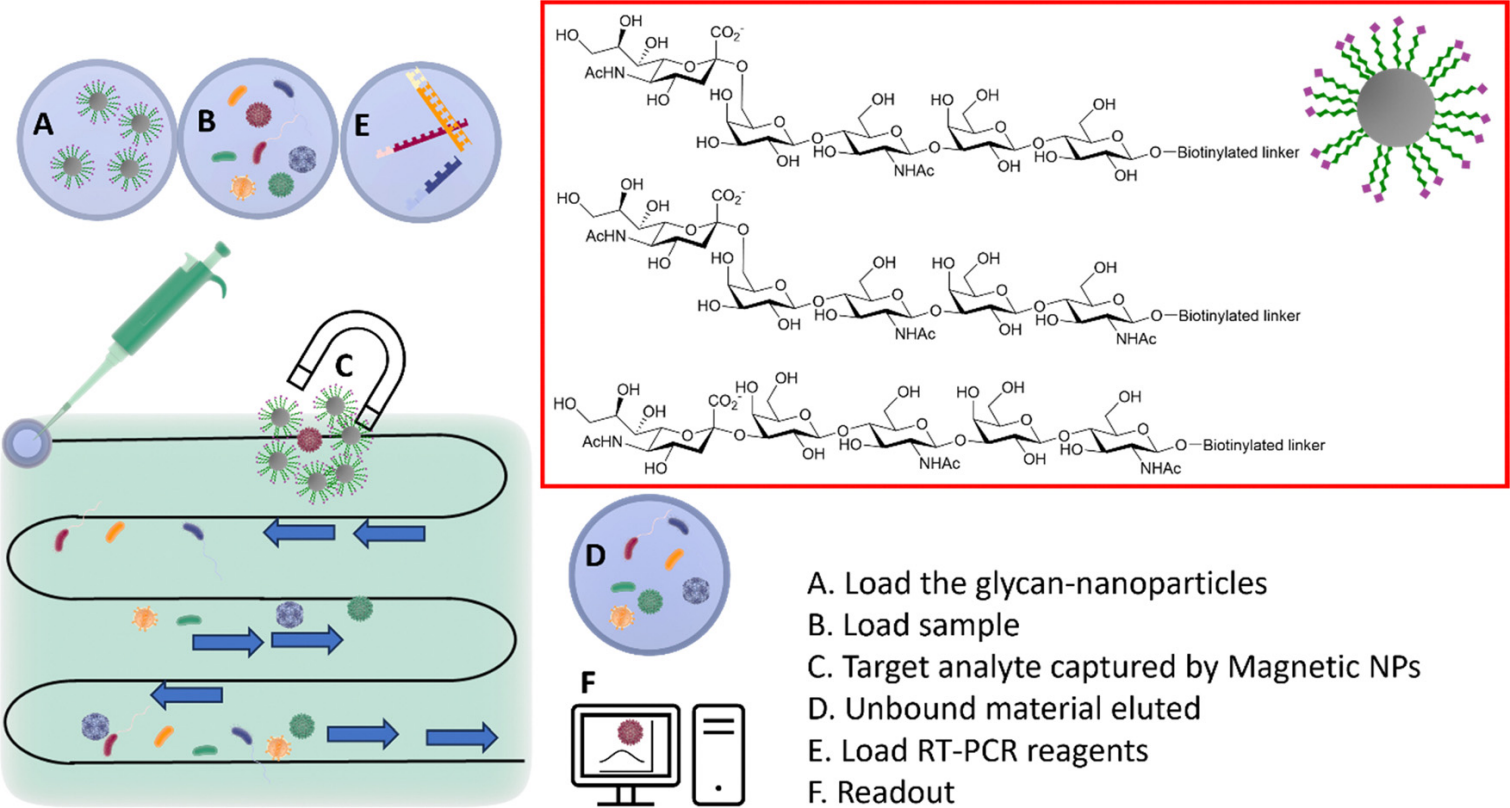
Integrated system combining microfluidic, magnetic nanoparticles and RT-PCR. (A) The glyco-nanoparticles are loaded into the microfluidic system; (B) the sample is then loaded in the microfluidic chip; (C) viruses binding to the specific glycan are captured by the magnetic nanoparticles; (D) the unbound material is eluted; (E) the RT-PCR reagents are loaded; (F) the readout provides information of the captured virus(es).^96^

The HA–sialic acid binding interaction can also be exploited in sample preparation/enrichment (Fig. 12),^96^ with a microfluidic chip system in tandem with glycan-coated magnetic beads to isolate influenza A viruses from complex biological samples, which were then analysed and quantified by RT-PCR.

### Colorimetric assays with sialic acid-containing glyconanoparticles and nanorods

**Fig. 13:**
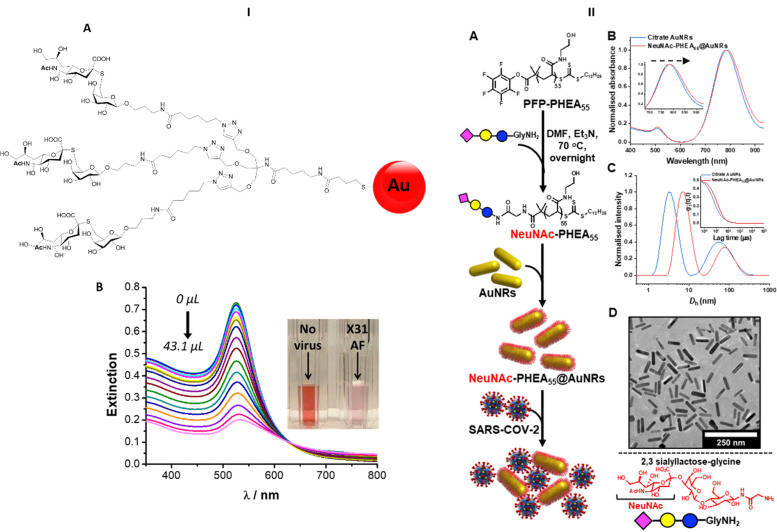
(I) Nanobiosensor for influenza detection exploiting the trimeric α-2,6-Neu5Ac-galactose ligand (A) conjugated to gold nanoparticles. The presence of the virus generates a colour change in the functionalised colloidal gold solution^97^ (B). (II) Gold nanorods functionalised with α-2,3′-Neu5Ac-lactose exploited for the rapid detection of SARS-CoV2.^98^

Nanobiosensors have been developed exploiting HA–sialic acid binding specificity. For instance, gold nanoparticles coated with trimeric α-2,6-thio linked Neu5Ac-Gal ligands bind selectively to human influenza virus H3N2-X31.^97^ A change in extinction of the colloidal suspension of gold nanoparticles upon recognition and binding to the virus occurred within 30 minutes after addition of the virus. Importantly, gold nanoparticles functionalised with α-2,6-thio-linked Neu5Ac-Gal ligand were able to discriminate between human and avian influenza viruses, allowing for species-specific virus detection (Fig. 13-I). These reagents were subsequently adapted for use in lateral flow tests for influenza viruses (Iceni Glycoscience, unpublished results).

The sialic acid binding properties of SARS-CoV2 have been exploited with gold nanorod plasmonic particles,^98^ which were functionalised with a PHEA polymer decorated with α-2,3′-Neu5Ac-lactose. These nanorods have dual absorption bands (520 nm and 785 nm), compared to a single band (520 nm) for gold nanoparticles, which offers advantage as the 520 nm band is impacted by sample matrix effects (Fig. 13-II). The glyco-nanorods successfully detected positives in clinical samples in a dose dependent manner, showing proof of concept application of the system.

### Electrochemical sensors presenting sialic acid

Another example of direct, label-free detection of influenza virus lies in the development of self-assembled monolayer-presented α-2,6′-Neu5Ac-lactose immobilised on gold electrodes.^99^ A significant signal is observed only upon binding of human influenza virus, showing the ability of the system to detect and discriminate between influenza virus strains. Importantly, in terms of hemagglutination titre (HAU), the sensitivity of this system (2^−4^ HAU) is much higher than that of immunochromatographic assay (2^2^–2^4^) or PCR (2^0^). Horiguhi *et al.*^99^ made a comparison of sensitivity, detection time and average cost with other detection methodology, summarised in Table 2.

**Table 2:** Detection system comparison between new and existing technologies

Detection system	Sensitivity (HAU)	Detection time	Cost (USD)
Label-free influenza virus detection (QCM^a^ detection)	2^−4^	10	35
Label-free influenza virus detection (electrical detection)	2^−6^	30	2
Immunochromatographic technique (ICT)	2^2^–2^4^	5–15	8–10
Detection of HA gene with PCR	2^0^	240	—
TLC virus overlay assay	2^8^–2^10^	—	—
Solid-phase virus binding assay	2^5^–2^10^	—	—

a Quartz crystal microbalance.

Another example of electrochemical sensor was presented by Hai *et al.*^100^ where a conducting polymer was functionalised with α-2,6′-Neu5Ac-lactose (or α-2,3′-Neu5Ac-lactose as control) showing specific recognition of H1N1 human influenza virus with a limit of detection of 0.013 HAU (for astandard immunochromatographic assay the LOD is 1.13 HAU).

### Sialic acid and SARS CoV2 detection on nitrocellulose strips

A rapid test has been reported for SARS-CoV2 detection that is based on sialic acid recognition in the form of a paper-based assay.^53^ Colloidal gold nanoparticles were functionalised with a poly *N*-hydroxyethyl acrylamide (PHEA) polymer equipped with either α-2,6′-Neu5Ac-lactose, α-2,3′-Neu5Ac-lactose or simply a Neu5Ac residue. The sample is deposited on a nitrocellulose strip and sialic-acid functionalised gold nanoparticles are eluted along the strip, generating a red spot in case of positive detection. Follow-through work^101^ tested the concept using clinical samples based on nasal swabs originating from COVID-19 positive patients showing the simple Neu5Ac decorated particles to have the best performance. In this instance, an additional silver staining step enhanced the limit of detection, with the unoptimized test achieving 85% sensitivity and 93% specificity, with cycle threshold (Ct) values as high as 25.

## Potential applications of sialic acids in protection against infection

The interplay between glycan structure, pathogen receptors and enzymes, and the host immune system lectin repertoire is key to both health and infection. With sialic acid as a dominant non-reducing terminal unit in many animal glycans, direct inhibition of its recognition or blocking or effecting the removal of this class of sugar has therapeutic potential in several ways. In addition, sialic acid-containing glycans produced naturally in the host can also have a protective role in preventing infection.

### Sialic acid-containing milk oligosaccharides

Human milk oligosaccharides (HMOs) are a biologically active component of breast milk that exert prebiotic effects (*i.e.* promote the growth and replication of commensal microorganisms), as well as other health promoting benefits to new-born infants.^102^ Over 200 different structures of HMOs have been identified in human breast milk,^103^ significantly more than are present in the milk of livestock and most primates.^104^ Roughly 50–70% of known HMOs are fucosylated, with *ca* 10–20% sialylated. The reverse is true for bovine and porcine milk oligosaccharides, where many milk oligosaccharides contain sialic acid.^105^ Numerous health benefits are thought to be associated with sialylated HMOs, with relevance to bacterial and viral infection, utilisation by gut commensals, direct modulation of the immune system and enhanced cognition and brain development. Animal milk oligosaccharides comprise the sialic acids Neu5Ac and/or Neu5Gc,^106^ including HMOs^107^ where the Neu5Gc is dietary derived due to the expression of an inactive CMP-Neu5Ac hydroxylase in man.^108^ The Neu5Gc from dietary sources, such as diary and red meat, is also found in human tissue, as indicated by circulating anti-Neu5Gc-antibodies and its incorporation into cancerous tumours.^109,110^

**Fig. 14:**
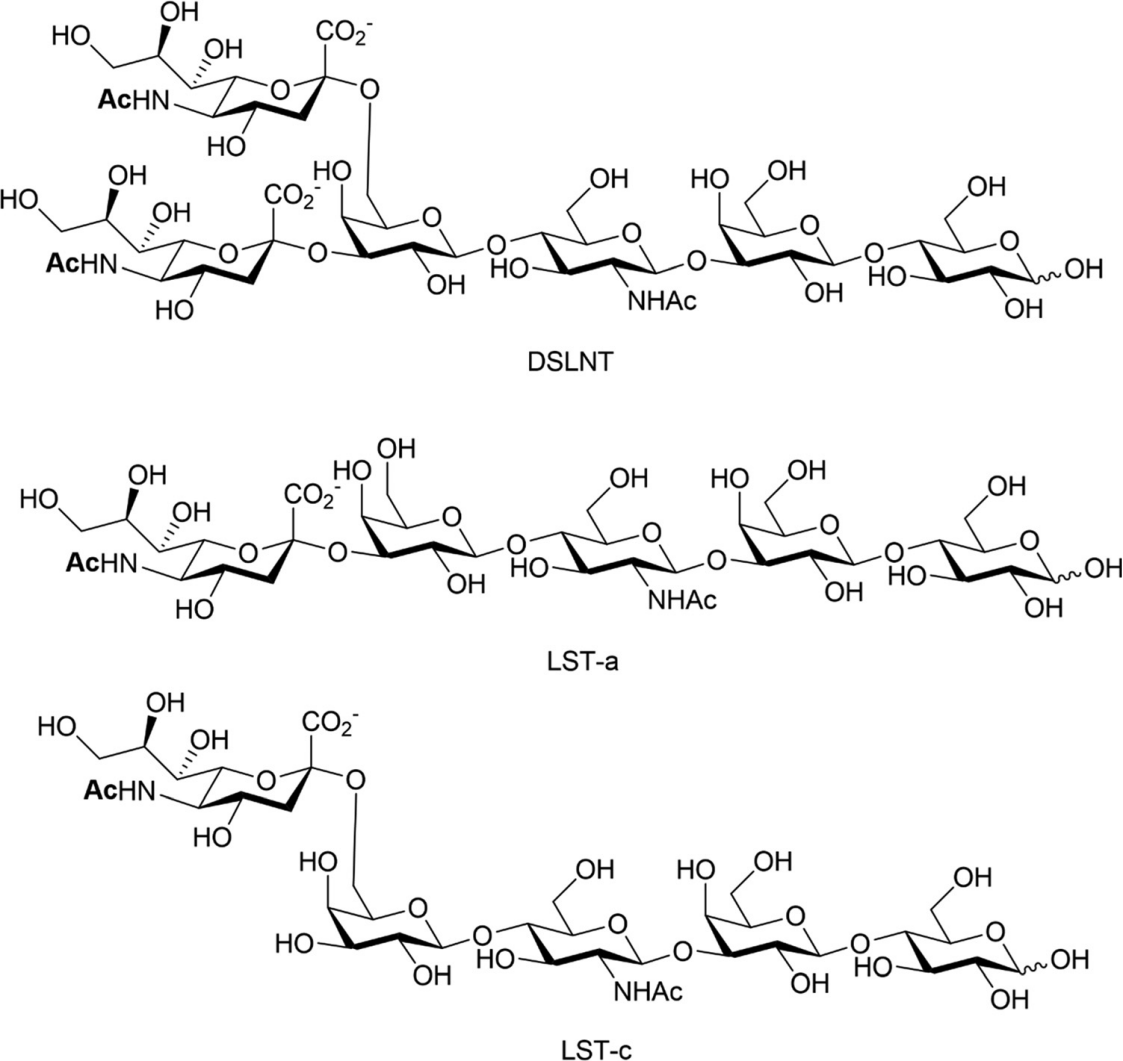
Chemical structures of HMO DSLNT and LST-a and LST-c.

HMOs are principally thought of as prebiotics – promoting the growth of beneficial microorganisms in the gut,^102^ particularly the commensals *Bifidobacteria spp*, *B. longum* and *B. bifidum.*^111^ Sialylated HMOs, specifically α-2,3′-Neu5Ac-lactose and α-2,6′-Neu5Ac-lactose which induce sialidase activity in *B. spp* and various *B. longum* strains, enable them to metabolise and grow on Neu5Ac and produce acidic fermentation products, lactate and short-chain fatty acids (SCFAs).^112^ Sialylated variants of lacto-*N*-tetraose exhibit antimicrobial activity against Group *B. Streptococcus*,^113^ presumably due to their ability to increase cellular permeability as seen in other studies on pooled HMOs.^114–116^ Additionally, previous work has shown that the hexasaccharide disialyllacto-*N*-tetraose (DSLNT, Fig. 14) contributes to the prevention of necrotising enterocolitis in a neonatal rat model.^117,118^ Acidic HMOs, particularly DSLNT, LS-tetrasaccharide a (LST-a,) and LS-tetrasaccharide c (LST-c) (Fig. 14), also have a pronounced effect on the modulation of intestinal epithelial cell maturation.^119^

Studies have indicated that sialylated HMOs can inhibit hemagglutination mediated by uropathogenic *E. coli* (UPEC) and enterotoxigenic *E. coli* (ETEC), thereby blocking bacterial adhesion.^120^ A similar process can be seen with *Helicobacter pylori*, where α-2,3′-Neu5Ac-lactose inhibits binding to the gastrointestinal epithelium^121^ and, in rotavirus, decrease replication.^122^ Acidic HMOs are also thought to possess antiviral properties. In *in vitro* hemagglutination inhibition assays of avian influenza viruses, α-2,3′-Neu5Ac-lactose exhibited antiviral properties. In addition, *in vivo* studies of pathogen-free chicken models treated with α-2,3′-Neu5Ac-lactose showed a reduction in symptoms when infected with H9N2 influenza virus, with the virus being completely eradicated within 24 hours.^123^

### Commensal intramolecular *trans*-sialidase

**Fig. 15:**
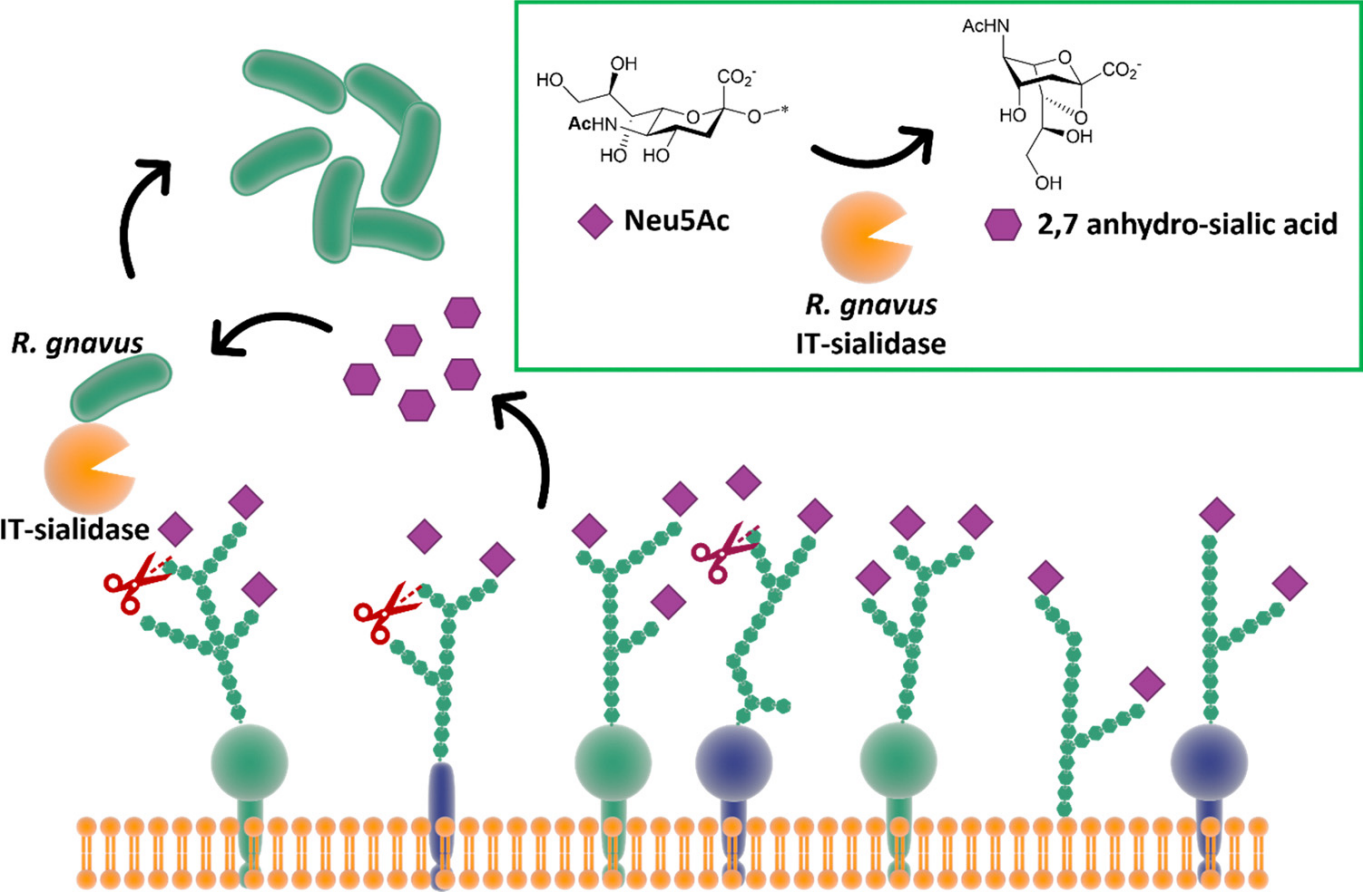
The IT-sialidase of *R. gnavus* cleaves sialic acid from host cell surface in the gut and rearrange it into 2,7 anhydro sialic acid, providing an advantage over other bacterial species in the gut able to metabolise the standard sialic acid.^126^

While most *trans*-sialidases have been studied from blood-borne trypanosomes,^64^ a novel intramolecular *trans*-sialidase has been identified in the commensal gut bacterium *Ruminococcus gnavus.*^124,125^ Studies revealed a new mode of action for this enzyme, which cleaves terminal α-2,3-linked sialic acid from human gut mucins and releases 2,7-anhydro-Neu5Ac instead of Neu5Ac. *R. gnavus* possesses a specific uptake mechanism for 2,7-anhydro-Neu5Ac that is not prevalent in nature, thus providing specific advantage for *R. gnavus* in scavenging sialic acid from sialylated glycans in the gut (Fig. 15).^126^ This in turn helps to maintain species balance and hence gut homeostasis, although a higher level of this intramolecular *trans*-sialidase enzyme has been found in patients with inflammatory bowel diseases.^127^

### Neu5Ac and small(er) molecule anti-influenza therapeutics

**Fig. 16:**
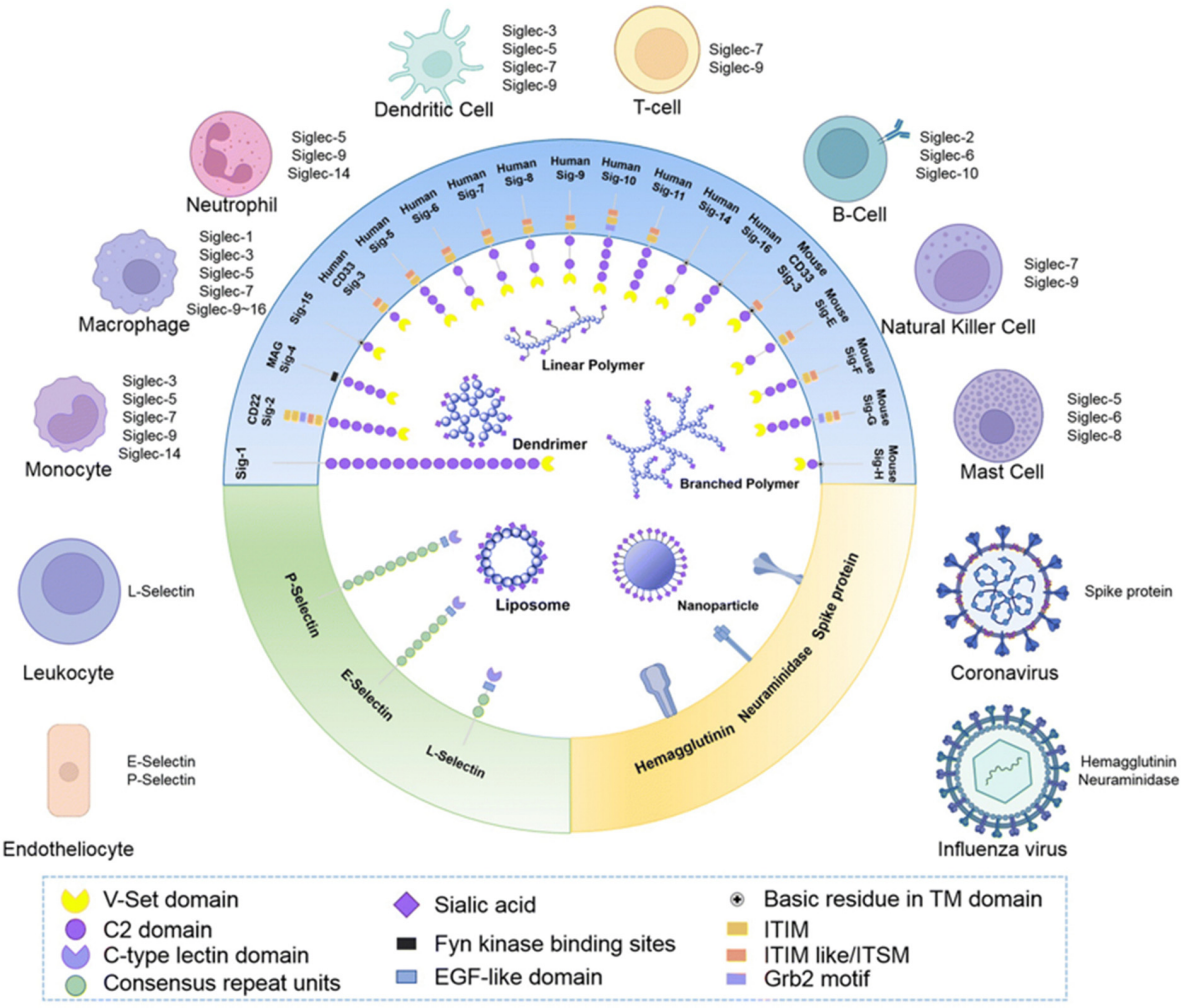
Schematic representation of how sialic acid and its derivatives have been exploited to generate multivalent materials to target receptors Siglecs, selectins and virus proteins to achieve immune modulation, targeted drug delivery and anti-virus treatments [reproduced with permission from *Biomaterial Science*, Royal Society of Chemistry, copyright 2013].^131^

Synthetic glycans,^128^ glycopeptides and glycopolymers^129,130^ have been informative with regards of the impact of glycan density and presentation (Fig. 16) on influenza virus infection. These studies demonstrate that glycan structure, valency and density (crowding) has a profound impact on NA binding and activity, and influenza virus adhesion and infectivity, respectively. Taken together, these studies suggest that multivalent inhibitors present obvious opportunities for therapeutic intervention.^131^ While this prospect has yet to be realised in the clinic, several studies have demonstrated approaches to high affinity ligands for the influenza surface-presented NA and, to a lesser extent, HA.

**Fig. 17:**
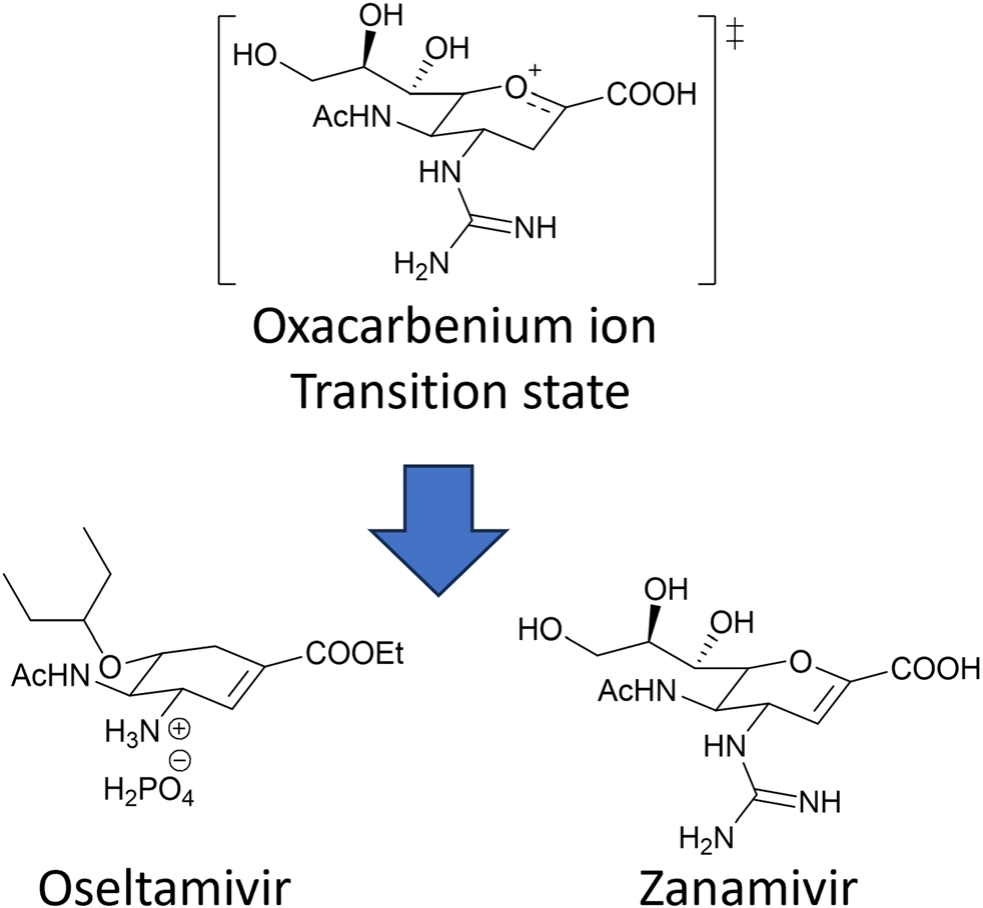
Chemical structure of the two NA inhibitors, Oseltamivir and Zanamivir, both mimic the oxacarbenium ion of the sialic acid intermediate formed during the NA action.

The coordinated action of NA and HA^132,133^ is responsible for binding of influenza virus to the sialylated cellular receptors, facilitating viral internalisation into host epithelial cells (HA)^38^ as well as daughter virion release (NA). Two currently approved anti-NA drugs, Zanamivir (Relenza) and Oseltamivir phosphate (Tamiflu) (Fig. 17), act as transition state analogue inhibitors targeting the active site of NA.

**Fig. 18:**
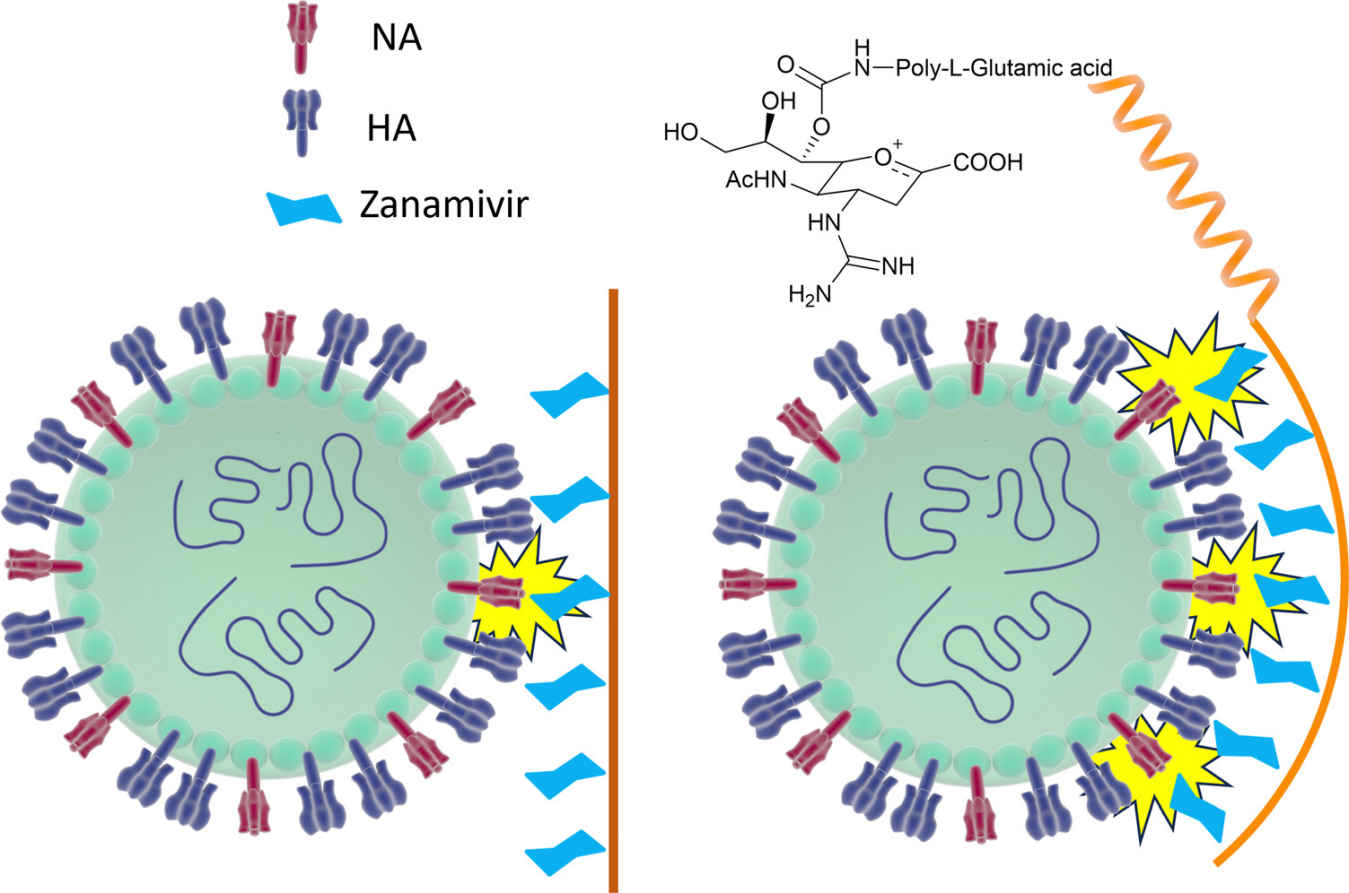
Schematic representation of how Zanamivir bound to a flexible polymer can maximise the interaction with surface NA and increase its binding strength.

However, mutations in and around the active site of the NA can lead to development of drug resistant strains resulting in the drugs being less effective in treatment and prevention of influenza virus infection. Weight *et al.*^134^ demonstrated that a multivalent polymer-bound Zanamivir binds 2000 times more strongly than its monomeric equivalent to the Zanamivir-resistant turkey/MN (Fig. 18).

To avoid drug resistance, a new strategy has been developed, where Zanamivir was covalently conjugated to a biocompatible water-soluble polymer and exhibited up to a 20 000-fold improvement in anti-influenza potency compared with the Zanamivir parent against human and avian viral strains, including both wild-type and drug-resistant mutants.^135^

**Fig. 19:**
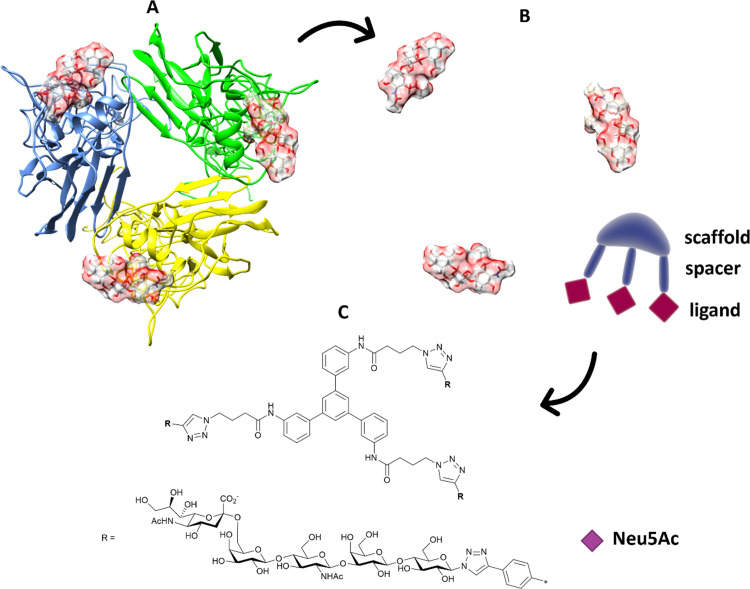
(A) The position of the ligands is elaborated from the 3D structure of the protein; (B) in terms of distance between ligands and orientation; the information is transferred to suitable trimeric structure (C) with scaffold and spacer to achieve the correct orientation and distance to get ligands binding simultaneously.^139^

Multivalent sialic acid-based HA lectin inhibitors^136,137^ can also provide anti-influenza activity by virtue of their ability to block virus–cell interactions. With HA ligands, this is more challenging that for NA ligands, as the monomeric ligand affinities for the latter are very much higher to start with. Nonetheless, polymer-stabilized sialylated nanoparticles can bind potently to and discriminate between influenza haemagglutinins.^138^ Inhibition of influenza A virus adhesion has been demonstrated for di- and tri-valent haemagglutinin inhibitors,^139^ (Fig. 19). By linking sialylated LacNAc units to di- and trivalent scaffolds, inhibitors were obtained that demonstrated >400-fold enhanced inhibition. Clearly, ligand presentation is central to achieving optimised affinity, as it is evident with natural glycan binding by influenza viruses. For instance, H3N2 viruses have specificity for α-2,6-sialylated branched *N*-glycans with at least three *N*-acetyllactosamine units (tri-LacNAc); the length of the glycan chain can be used to target enhance discrimination between virus strains.^140^

### Virucidal sialic acid materials

**Fig. 20:**
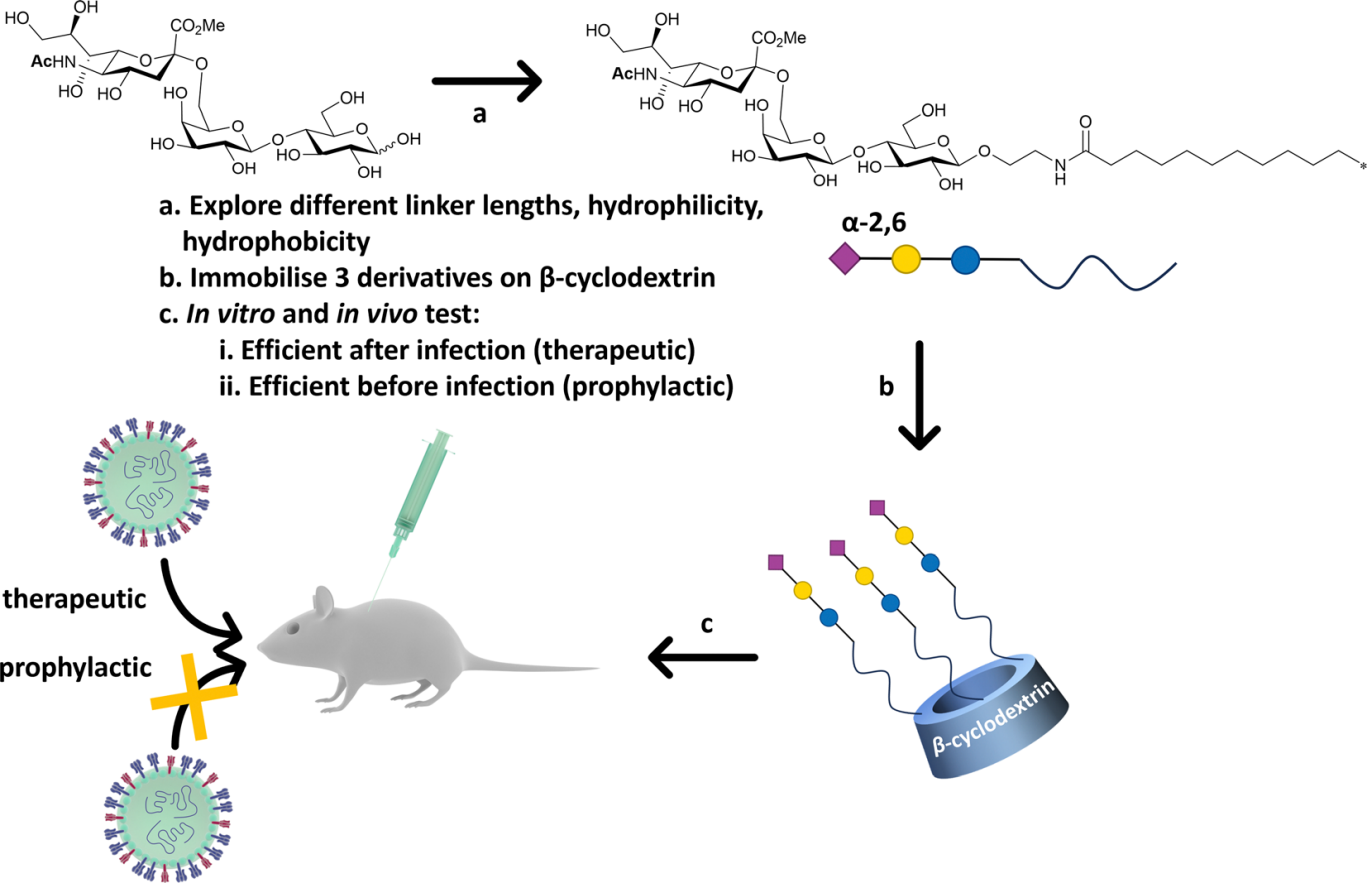
β-Cyclodextrin was used as scaffold to immobilise α-2,6′-Neu5Ac-lactose decorated with different linker. The most efficient configuration in terms of therapeutics and prophylactic activity was obtained with a hydrophobic linker. The construct was efficient in both *in vitro*, *ex vivo* and *in vivo* (mice) experiments against human influenza H1N1 infection.^141^

Precise presentation of sialic acid is an important factor in achieving inhibition of influenza virus infection. Decorating a β-cyclodextrin scaffold with three copies of sialic acid derivatives (Fig. 20), either α-2,6′- or α-2,3′-Neu5Ac-lactose, achieves effective inhibition of human and avian influenza virus infection, respectively.^141^ The authors carefully examined the impact of the sialic acid linker used to immobilise the carbohydrate moiety on the cyclodextrin scaffold, with a hydrophobic linker being more effective than a hydrophilic one. Significantly, the cyclodextrin–sialic acid constructs showed excellent virucidal properties – *i.e.* the compounds disrupted the virus structure, rather than just binding to HA. They also proved to be effective both as prophylactic agent when administered pre-infection, and as a therapeutic when administered post-infection in mice.

### Multivalent sialic acid-binding lectins block recognition of host cells by influenza virus

**Fig. 21:**
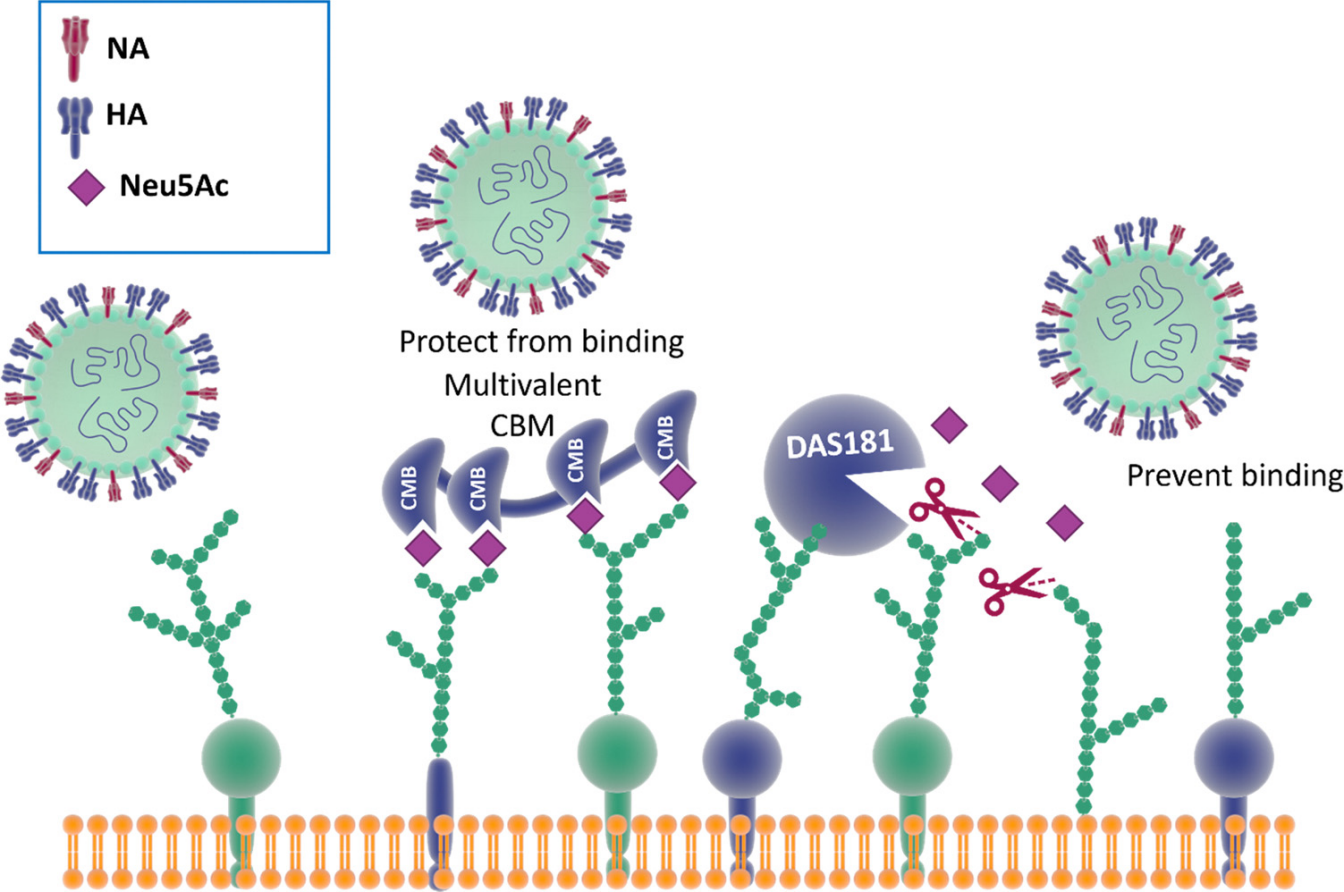
The binding of CBMs to sialic acid act as a shield, preventing the virus from bind to the same receptors; DAS181, instead, prevents the binding of the virus by cleaving the terminal sialic acid effectively destroying the surface cell receptors.^142,147^

Reports show that masking host cell sialic acid receptors with engineered multivalent sialic acid-recognising carbohydrate binding modules (CBMs) (Fig. 21) provided protection to mice against the 2009 pandemic H1N1 influenza virus^142^ and the influenza A (H7N9) virus.^143^ The authors suggested that this host-targeted approach could provide a front-line prophylactic that has the potential to protect against any current and future influenza virus and possibly against other respiratory pathogens that use sialic acid as a receptor. Furthermore, the same CBM constructs were shown to possess immunoregulatory properties,^144,145^ supporting the notion that they could be used not only to protect from on-going disease, but that they could modulate immune responses to prevent future infections and potentially find application as adjuvants^146^ for vaccines.

### Therapeutic sialidase-mediated removal of sialic acid prevents influenza infection

An alternative strategy^147^ employed to block influenza infection targets the host cell sialic acids, rather than the two viral surface proteins HA and NA discussed above, has been reported. DAS181 (Fludase) is composed of a sialidase catalytic domain, cleaving α-2,6- as well as α-2,3-sialic acid, fused with a cell surface-anchoring sequence, which destroys essential sialic acid receptors and consequently blocks viral adhesion (Fig. 21). *In vitro* assays of laboratory strains and clinical isolates of influenza A and B viruses showed EC_50_ values range from 0.04 to 0.9 nM. In another similar study, DAS181 showed strong inhibition against a panel of Oseltamivir resistant H1N1 using plaque number reduction assay on MDCK cells.^135^ Currently, DAS181 has completed pre-clinical development and has entered clinical Phase I and Phase II trials, with the latest clinical data showing that DAS181 significantly reduces viral load in participants infected with influenza virus, thus justifying future clinical development of this novel host-directed therapy.^148^

### Neuraminidase inhibitor-mediate immunotherapy for influenza infection

**Fig. 22:**
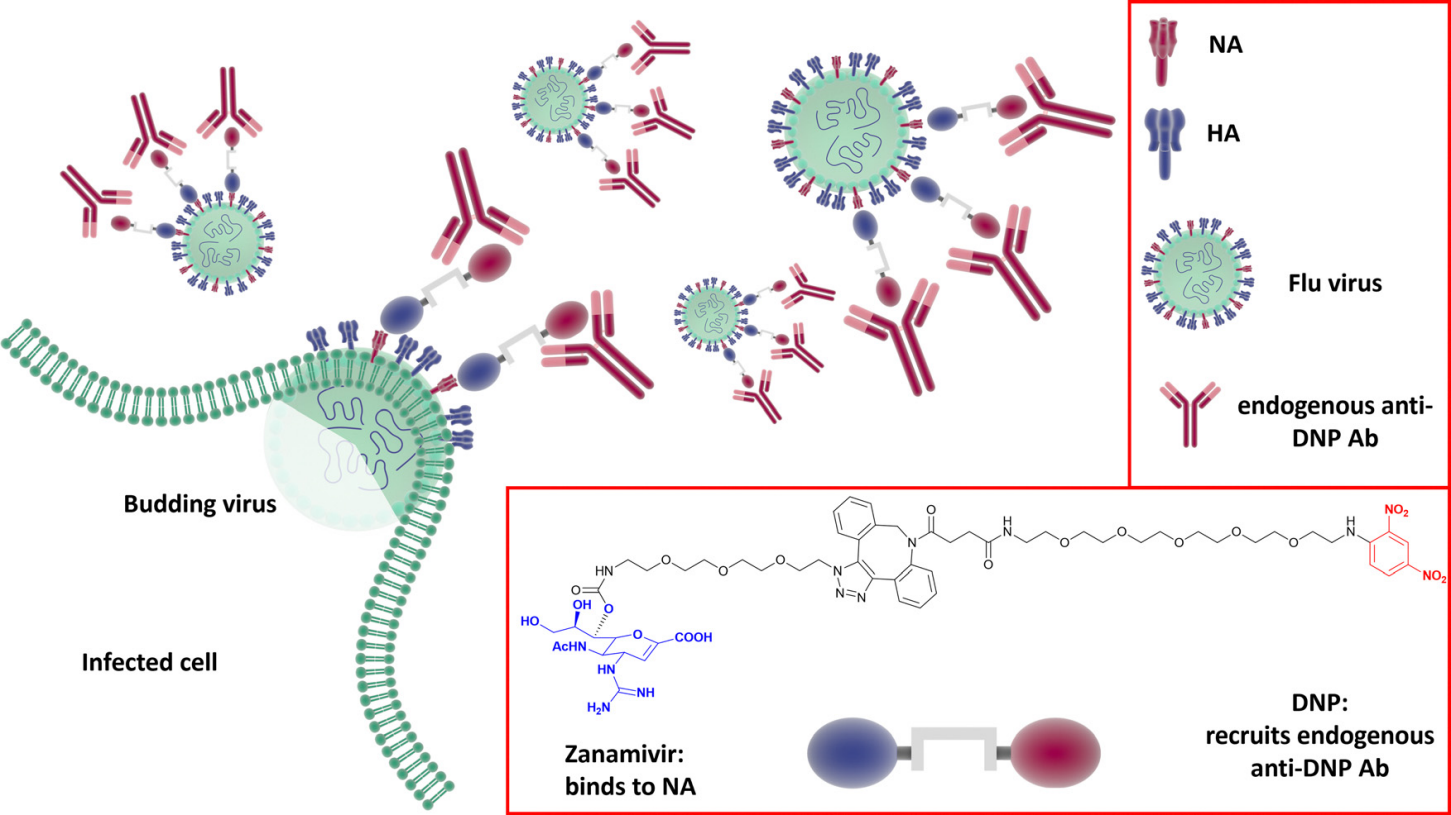
The dual Zanamivir-dinitrophenyl conjugate binds to surface NAs of the virus inhibiting the neuraminidase activity and suppressing virus budding from the host cell. The dinitrophenyl (DNP) hapten is highly immunogenic and recruits endogenous anti-DNP antibodies both on the virus-free and the virus-infected cell resulting in their opsonization and the consequent immune-mediated clearance.^149^

Harnessing the host immune response to specifically target influenza virus presents a novel approach to anti-viral therapy. A synthetic bifunctional small molecule was prepared by conjugating the NA inhibitor Zanamivir with the highly immunogenic dinitrophenyl group,^149^ which specifically targets the surface of free virus and viral-infected cells (Fig. 22). This approach has dual function, in that the Zanamivir blocks daughter virion release from host cells, while the primed immune response serves to attack and clear virus from the body. In relation to severe infections, this therapeutic regimen remains effective up to three days post lethal inoculation, suggesting that it may be useful for infections refractory to established therapies.

## Summary

Given the ever-increasing demonstration of roles for glycans in immune health and disease,^150^ with impact for infection and the prevention thereof, the need for much further investigation of the glycobiology of cell surfaces is called for. In addition, in terms of therapeutic intervention, biopharmaceuticals, which are often sialylated, are very much to the fore at present. The glycosylation, and in particular sialylation^151^ thereof, is crucial to the optimisation of efficacious, cost-effective, and safe medicines.^152^ As far back as 2001, with reference to glycoscience it was projected that “Cinderella's coach is ready”.^153^ In the intervening period fundamental glycomics studies continue to advance at pace,^154^ with sialic acids central to the investigation of infection studies. As a field, however, glycomics lies some way behind other omics topics (Table 3), highlighting the challenge, the opportunity and the unmet need that glycoscience presents.

**Table 3:** Comparison of the volume of ‘omics’ publications by discipline. Web of Knowledge literature search 08/07/2023. [inspired by presentation from Ole Hindsgaul]

Topic	Number of publications to date
Genomics	203 551
Proteomics	123 055
Metabolomics or metabonomics	59 203
Lipidomics	9713
Glycomics	3790

Translational impact for sialic acids faces a number of challenges going forwards. While the scalable enzymatic synthesis of sialic acid^155–158^ and sialylated glycans^159–161^ has been achieved, the correct presentation of this key sugar recognition element is critical to achieving physiologically or therapeutically relevant biological recognition. For instance, the valency of NeuAc presentation^97^ as well as secondary interactions from the glycan chain to which it is attached^140^ can have a profound impact on target engagement. In addition, the efficiency of glycan recognition is context dependent, in that monovalent affinity does not directly correlate with polyvalent avidity.^162^ So consideration needs to be given not only to glycan structure, but also to the assay format used when considering cell adhesion events, for instance.

A further challenge lies in the myriad of sialic acid modifications found in nature, some of which (*e.g.* sialylation) are labile^163^ or prone to intramolecular *O*-acetyl migration,^164^ but which may have a profound effect on enhancing or masking sialic acid recognition events. Further still, sialic acids are key players in the immune system, where true physiological effect and therapeutic potential can only be achieved through *in vivo* studies – and all animals are not equal in glycoimmunology.^165,166^ Nonetheless, as highlighted in this article, substantial advances are being made to open up sialic acid biology and therapeutics.

The central role of sialic acids in infections is clear cut and there has been longstanding success with inhibitors of sialic acid metabolism in the prevention of influenza infection, in particular. The current state of play provides much in the way of foundational tools and initial leads, leading to much optimism about the prospect of a rich future of sialic acid-related diagnostics, prophylactics and therapeutics going forwards.

## Conflicts of interest

Iceni Glycoscience has active programs on the recognition of sialic acids for the development of diagnostics and therapeutics for infectious diseases.

## Supplementary Material
